# Supramolecular helicity dependent osteogenesis and angiogenesis crosstalk of periodontal ligament stem cell

**DOI:** 10.1016/j.bioactmat.2025.12.057

**Published:** 2026-01-02

**Authors:** Zhuohang Deng, Meijun Li, Yifan Wang, Wenjing Li, Zijian Gong, Peiwen Liao, Shengzhen Luo, Minghua Liu, Xuliang Deng

**Affiliations:** a School of Stomatology, Hebei Medical University, Shijiazhuang, 050011, China; b Beijing Key Laboratory of Biomaterials for Oral Disease, Department of Geriatric Dentistry, Peking University School and Hospital of Stomatology, Beijing, 100081, China; c Beijing National Laboratory for Molecular Science, Institute of Chemistry, Chinese Academy of Sciences, Beijing, 100190, China; d Center for Drug Research and Evaluation, National Infrastructures for Translational Medicine, Peking Union Medical College Hospital, Chinese Academy of Medical Sciences, Beijing, 100730, China; e Department of Prosthodontics, The First Clinical Division, Peking University School and Hospital of Stomatology, Beijing, 100034, China; f Peking University Health Science Center and Hospital of Stomatology, Beijing, 100081, China

**Keywords:** Chiral biomaterials, Chiral self-assembly, Osteogenic-angiogenic coupling, Periodontal ligament stem cells, Bone regeneration, Mechanotransduction

## Abstract

The two pillars supporting effective tissue regeneration are multipotent stem cells and matrix materials that direct differentiation. The chirality of the extracellular matrix is a key structural characteristic that affects stem cell fate. However, little is known about the effects of either molecular chirality or supramolecular helicity on the differentiation of periodontal ligament stem cells (PDLSCs), a safe and easily accessible stem cell source. Here, we constructed fibrils through the co-assembly of chiral amino acid derivative enantiomers (*l*/*d*-GC18) and a bridging pyrazine molecule. The helicity of the fibrils depends on both the molecular chirality of the amino acid and the stoichiometric ratio of the two components. Our results showed that molecular chirality and supramolecular helicity can act synergistically, with the left-handed fibrils assembled from *l*-GC18 and pyrazine promoting osteogenic differentiation of PDLSCs *in vivo*. Moreover, the chiral fibrils effectively promoted bone regeneration in both the calvarial and alveolar bone defect models. Interestingly, it was observed that left-handed fibrils induced integrin-dependent osteogenic differentiation, which in turn stimulated Piezo1-mediated, Vascular Endothelial Growth Factor (VEGF)-driven angiogenesis. These findings thus provide a blueprint for harnessing PDLSCs in next-generation regenerative therapeutics.

## Highlights

• We developed a bioinspired chiral amino acid-derived system that self-assembles into supramolecular fibrils with programmable helicity, characterized using AFM and Cryo-EM.
• Left-handed helicity selectively enhances osteogenic differentiation and pro-angiogenic paracrine secretion in periodontal ligament stem cells (PDLSCs), thereby coupling osteogenesis with angiogenesis.
• Mechanistically, chiral topology activates integrin α5β1-mediated mechanotransduction, triggering dual signaling pathways: (1) FAK-ERK-YAP/RUNX2 for osteogenesis and (2) Piezo1-Ca²⁺-HIF1α-VEGF for angiogenesis.
• The left-handed fibril/PDLSC construct promotes robust bone regeneration in critical-sized calvarial and alveolar defect models.
• This work establishes a chirality-driven biomaterial design strategy that leverages mechano-chemical crosstalk to program stem cell microenvironments for synergistic tissue regeneration.

## Graphical abstract

**Figure:**
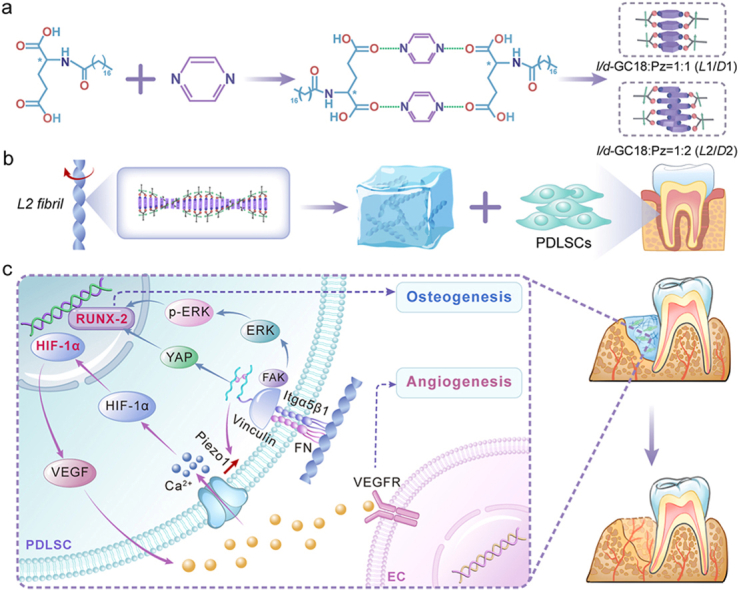

## 1 Introduction

**Scheme 1:**
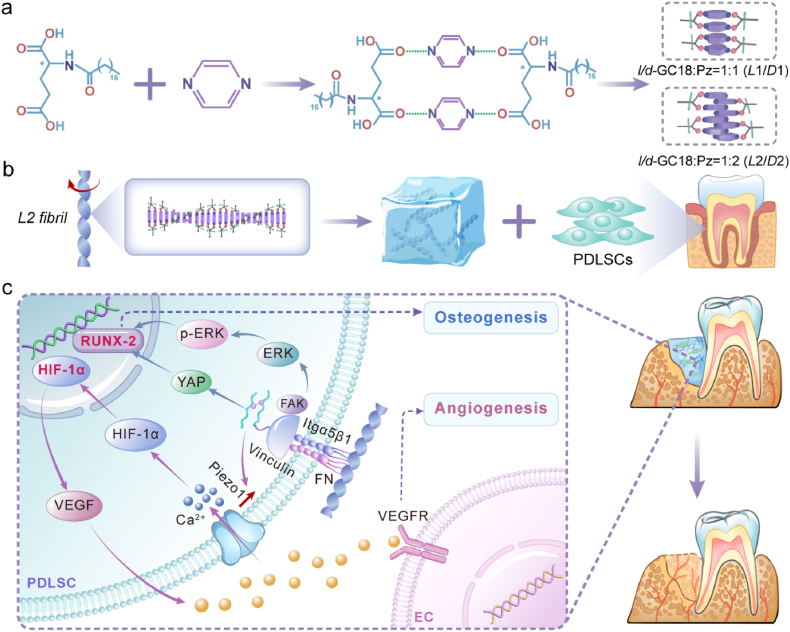
Schematic illustration of the supramolecular helicity-dependent crosstalk between osteogenesis and angiogenesis in PDLSCs. a) Molecular structure of *l*/*d*-GC18 and its self-assembled nanofibrils. b) Nanofibrils self-assemble into a semi-transparent sol suspension, which is used to precondition periodontal ligament stem cells (PDLSCs) before their implantation into rat alveolar bone defects. The left-handed helical nanofibrils (*L*2) demonstrate effective osteogenic and angiogenic performance. c) *L*2 fibril-mediated mechanotransduction pathways. Stereospecific activation of integrin α5β1 through chiral topological recognition activates the FAK–ERK–YAP/RUNX2 signaling axis to drive osteogenesis, as well as trigger Piezo1-mediated Ca^2+^influx to potentiate HIF1α-dependent VEGF secretion, which in turn promotes angiogenesis.

Tissue engineering is a promising approach for tackling degenerative diseases and injuries such as type 1 diabetes [1], Alzheimer's disease [2], and bone defects [3], which have achieved remarkable improvements in the physical health of patients. The two key components supporting effective tissue engineering are stem cells and matrix materials that direct differentiation [4], which raise concerns about the ethical source of stem cells and effective techniques for promoting their lineage determination, respectively [5,6] (see Scheme 1).

Chirality is a universal phenomenon in nature and profoundly influences the properties of both natural biostructures [7] and synthetic materials [[8], [9], [10]]. Designing specific chiral structures to direct stem cell lineage diversification has emerged as a key frontier in tissue engineering and regenerative medicine. Spatial matching to specific molecular targets plays a pivotal role in the recognition of both material supramolecules and drug molecules, with morphological chirality being one of the key properties [9,11]. Our previous studies have demonstrated that chiral fibril-doped extracellular matrix can modulate bone marrow stem cell lineage commitment through mechanoresponsive signaling pathways [12]. Previously, we characterized chirality by optical methods such as CD, but the morphological characterization is unsatisfactory due to limitations in material systems and characterization techniques [12,13]. Moreover, recent studies have shown that optical chirality does not correspond to morphological helicity [14,15]. Hence, the role of supramolecular helicity of the extracellular matrix remains unknown.

Periodontal ligament stem cells (PDLSCs), a mesenchymal stem cell subpopulation residing in the periodontal ligament, can be readily isolated from both deciduous and permanent teeth [16]. With minimal ethical concerns, PDLSCs represent a promising cell source due to their immunocompatibility and accessibility, serving as a viable alternative to induced pluripotent stem cells or bone marrow-derived mesenchymal stem cells [17]. Accordingly, research in periodontal regeneration has evolved from simple cell transplantation towards creating biomimetic microenvironments and intelligent modulatory strategies. Current efforts focus on several key areas, including fabricating biomimetic extracellular matrices via advanced manufacturing technologies [18,19]; developing periodontium-mimicking models with stem cell manipulation [20]; and exploring novel regulatory signals and pathways [[21], [22], [23]]. Despite these advances, how molecular chirality and supramolecular helicity influence PDLSC differentiation remains a critical and unresolved scientific question.

The chirality of supramolecular assemblies is typically influenced by the molecular chirality of the constituent molecules and the conditions of assembly [24]. Hence, under physiological conditions, regulating the chirality of supramolecules assembled by molecules with predefined molecular chirality remains challenging. The DNA origami technique, with a programmable double helix that relies on hydrogen bonding pairing [25,26], offers a potential solution by enabling precise structural control at the nanoscale level via finely tuned binding interactions. Taking inspiration from nature, we synthesized chiral amino acid derivatives with a head group inclined to form hydrogen bonding pairs and long alkyl tails to promote self-assembly. This is designed to construct paired stands of supramolecular fibrils with programmable hydrogen bonding. The chiral structures were clearly and uniformly determined in this study, which is key to delineating the relationship between chiral nanostructure and PDLSC lineage determination.

## 2 Results and discussion

### 2.1 Design and characterization of nanofibrils with stoichiometry-dependent helicity

**Fig. 1:**
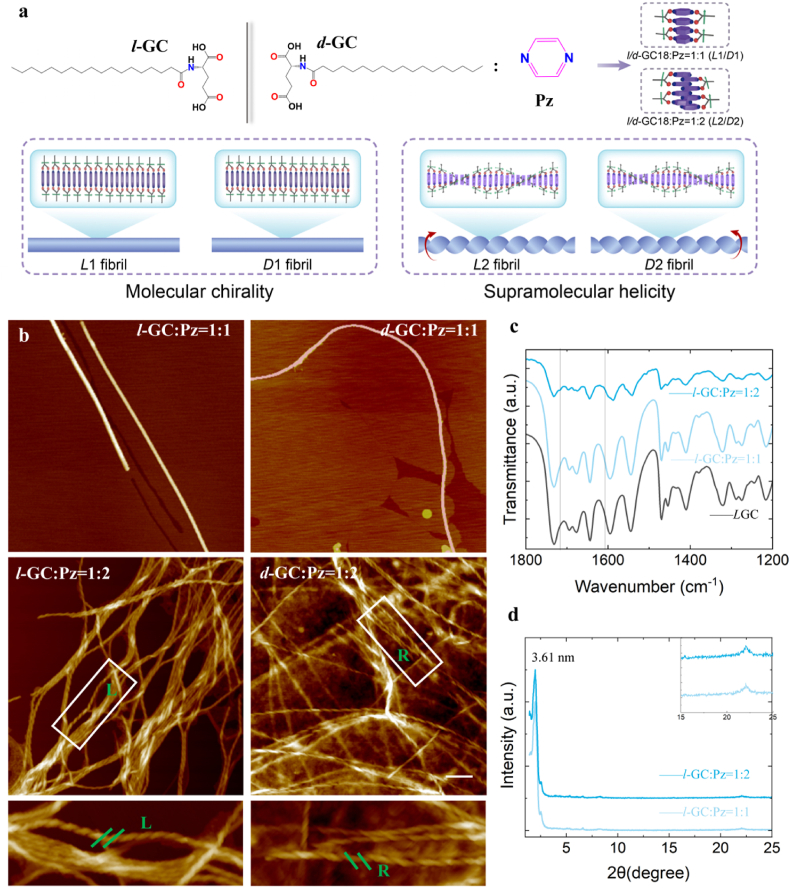
**Molecular design and structural characterization of the nanofibrils with stoichiometry-dependent helicity.** a) Molecular structures of *l*/*d*-GC18 and schematic illustration of their co-assembly with Pz into nanofibrils. At a 1:1 M ratio of *l*-GC to Pz, non-helical *L*1 fibrils formed, exhibiting chirality confined to the molecular level. In contrast, a 1:2 ratio of *l*-GC to Pz yielded left-handed helical *L*2 architectures, demonstrating emergent chirality at the supramolecular level. Similarly, a 1:1 ratio of *d*-GC to Pz produced non-helical *D*1 fibrils with molecular-level chirality, while a 1:2 ratio resulted in right-handed helical *D*2 superstructures, exhibiting hierarchical chirality spanning molecular and supramolecular scales. b) AFM images of nanofibrils co-assembled from *L*GC (left) and *D*GC (right) at molar ratios of 1:1 (top) and 1:2 (bottom). c) FT-IR spectra of *L*GC fibrils co-assembled with increasing concentrations of Pz. d) XRD pattern of *L*GC fibrils.

We designed a pair of glutamic acid derivative enantiomers (*l*/*d*-GC18) to synthesize fibrils with programmable supramolecular helicity in aqueous solution. The chiral hydrophilic head group (glutamic acid) and the hydrophobic tail (octadecyl) are connected by an amide bond, which is predisposed to form intermolecular hydrogen bonds (H-bonds, Fig. 1a, green arrows). The *l*/*d*-GC18 molecules tend to form strands driven by hydrogen bonding and hydrophobic interactions. To bridge these strands into a double helix, we introduced pyrazine molecules. The pyrazine ring contains two nitrogen atoms at para positions, both of which can form an additional set of hydrogen bonds with the carbonyl groups of the glutamic acid residues.

The glutamic acid derivatives (*l*/*d*-GC18) were synthesized in two steps from commercially available diethyl *l*/*d*-glutamate hydrochloride and stearic acid with high overall yields (Supplementary Scheme 1). The purified *l*/*d*-GC18 powder was dispersed in an aqueous pyrazine (Pz) solution at molar ratios of 1:1 or 1:2. The suspension was heated and thoroughly sonicated, then allowed to cool naturally to room temperature. After cooling, the translucent suspension formed a hydrogel.

The morphology, particularly the helicity of the assemblies (1:1), was characterized by atomic force microscopy (AFM), and corroborated by Cryo-electron microscopy (cryo-EM) in detail. When *l*/*d*-GC18 and Pz were present in equal molar amounts (1:1), the assembled fibrils displayed no detectable helicity (Fig. 1b) as revealed by both cryo-EM and AFM (Supplementary Figs. 1 and 2). Circular dichroism (CD) spectra of the *L*-GC18 (*L*1) and *D*-GC18 fibrils (*D*1) exhibited opposite peaks at the absorption wavelength of Pz (Supplementary Fig. 3). These results thus indicate that the molecular chirality of glutamic acid derivatives can be transferred to Pz, but not to the assembly collectively at equal molar ratios.

When the number of Pz molecules is twice that of GC18 molecules (2:1), the fibrils are chiral with helicity dependent on the chirality of GC18. As shown by the AFM results, *l*-GC18 self-assembled into left-handed fibrils, while *d*-GC18 self-assembled into right-handed fibrils. The CD spectra of *L*-GC18 (*L*2) and *D*-GC18 fibrils (*D*2) also showed opposite peaks compared to the absorption peak of Pz (Supplementary Fig. 3). These results thus indicate that GC18 and Pz co-assembled into fibrils, the molecular chirality of glutamic acid derivatives can be transferred to Pz, and more importantly, to the supramolecular assemblies when the quantity of Pz is twice that of GC.

Hence, we successfully synthesized nanofibrils by the co-assembly of the artificially designed glutamate acid derivatives. Interestingly, the supramolecular helicity depended on both the molecular chirality of the glutamic acid derivatives and the molar ratio between the chiral molecule and the bridging molecule.

To reveal the packing and molecular arrangement of the stoichiometry-dependent helicity of these nanofibrils, we conducted Fourier-transform infrared (FT-IR) spectroscopy. The FT-IR spectra of the nanofibrils showed both asymmetric and symmetric CH_2_ stretching vibrations at 2918 and 2850 cm^−1^ (Supplementary Fig. 4), suggesting a similar zig-zag conformation [27] of alkyl chains both in the *L-* and *D*-GC fibrils. GC molecules were designed to form H-bonds through the carboxyl group on Pz to facilitate the co-assembly. FT-IR spectra provided information to identify the H-bonding state. The stretching vibration band of the carbonyl group (*ν*-C=O at 1595 cm^−1^) on GC fibrils weakens with the appearance of peaks at 1599 and 1720 cm^−1^ with the addition of Pz (*L*2 fibrils), indicating the formation of hydrogen bonds between the carbonyl group and Pz.

Fig. 1d displays the X-ray diffraction (XRD) patterns of the nanofibrils. According to Bragg's equation, the d-spacing was estimated to be 3.61 nm, suggesting a bilayer structure formed by GC molecules. The diffraction peaks at 2θ values of 22.05° (Fig. 1d, insert) gave d-spacing values of 0.42 nm, indicating the π-π stacking of Pz. The FT-IR and XRD results showed collectively that a composite bilayer was formed, in which Pz molecules were packed in an orderly manner between the *l-* or *d-* GC molecules through hydrogen bonding.

### 2.2 Supramolecular helicity and molecular chirality orchestrate Osteogenic–Angiogenic coupling *in vivo*

**Fig. 2:**
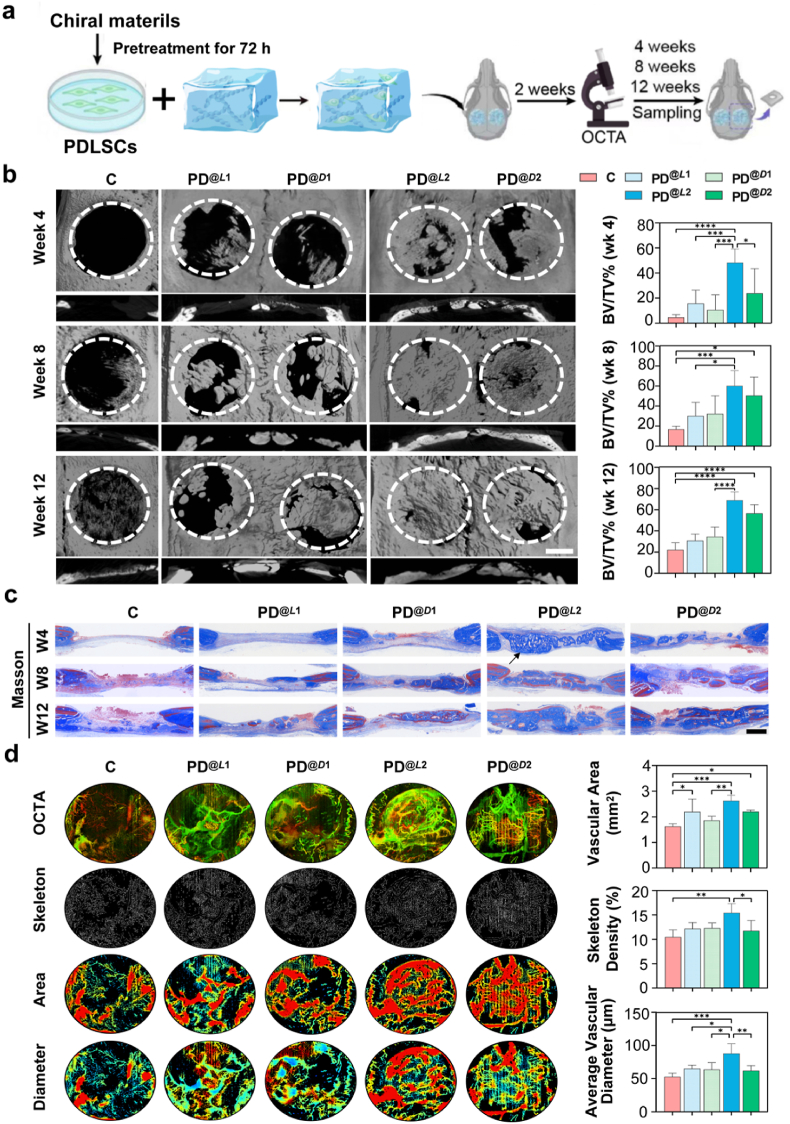
**Helicity-dependent Bone and Vascular Regeneration.** a) Schematic illustration of the experimental procedure for bilateral calvarial defect implantation using PDLSCs pretreated with chiral nanofibrils (defect diameter = 5 mm; *n* = 6/group). b) Representative micro-CT images of bone regeneration in rat cranial defects at 4, 8, and 12 weeks after treatment (scale bar: 500 μm). PDLSCs treated with the left-handed fibril (PD^@*L*2^) showed the most abundant new bone formation. The right panel presents the quantitative analysis of the bone volume to total volume (BV/TV) ratio in the newly regenerated tissue (Data are presented as mean ± s.d. (*n* = 6); ∗*P* < 0.05, ∗∗*P* < 0.01, ∗*P* < 0.001; one-way ANOVA with Holm-Šidák post hoc test). c) Representative histological sections of the defect site with Masson's trichrome staining (collagen maturation; blue: mineralized, red: osteoid) (scale bar: 500 μm). d) OCTA vascular mapping at day 14 and quantitative analysis of skeleton density, vascular area, and average vascular diameter (Data are presented as mean ± s.d. (N = 6); ∗*P* < 0.05, ∗∗*P* < 0.01, ∗*P* < 0.001; one-way ANOVA).

The four types of nanofibrils co-assembled from *l-* or *d-*glutamic acid derivatives and pyrazine demonstrated excellent biocompatibility with PDLSCs, as confirmed by CCK-8 and live/dead assays (Supplementary Fig. 11), making it feasible to evaluate their biomedical applications *in vivo*. We first preconditioned PDLSCs for 3 days separately with each of the four nanofibrils: i.e., the straight (PD^@*L*1^) and left-handed (PD^@*L*2^) fibrils co-assembled from *l*-GC18 and pyrazine, and the straight (PD^@*D*1^) and right-handed (PD^@*D*2^) fibrils co-assembled from *d*-GC18 and pyrazine. The pre-conditioned cells were then harvested, mixed with their corresponding fibrils, and implanted into fresh rat calvarial defects (Fig. 2a).

Bone regeneration at the defect site was evaluated at 4, 8, and 12 weeks after implantation of the preconditioned PDLSCs. Micro-CT analysis revealed that both left- (PD^@*L*2^) and right-handed (PD^@*D*2^) fibrils induced significantly higher bone volume fractions (BV/TV) than both their linear counterparts (PD^@*L*1/*D*1^) and negative controls (*P* < 0.05; Fig. 2b). Notably, the PD^@*L*2^ group exhibited the most vigorous osteogenic performance among all groups. Moreover, the defect areas in the PD^@*L*2^ group showed enhanced bone deposition, accompanied by aligned trabecular and collagen fibers, as observed in both the H&E and Masson's trichrome staining (Fig. 2c, Supplementary Fig. 5a). Immunofluorescence staining further showed significant upregulation of osteogenic markers in the regenerated tissue at the defect site in the PD^@*L*2^ group, including bone morphogenetic protein 2 (BMP2), runt-related transcription factor 2 (RUNX2), and osteopontin (OPN) (Supplementary Fig. 5b). These results thus indicated that the left-handed fibrils assembled from *l*-GC18 were the most effective in inducing osteogenesis of PDLSCs. This suggests a synergistic effect between supramolecular helicity and molecular chirality in promoting PDLSC-enabled bone regeneration. Notably, PDLSCs, a cell lineage traditionally considered to have moderate osteogenic potential [28], exhibited bone formation rates comparable to those of bone marrow mesenchymal stem cells (BMSCs), as reported previously [29].

Interestingly, optical coherence tomography angiography (OCTA) on day 14 revealed significantly enhanced vascularization parameters in the PD^@*L*2^ group compared to controls, including skeleton density, vascular area, and vessel diameter (P < 0.05; Fig. 2d), indicating that the left-handed fibrils effectively promoted angiogenesis. This pro-angiogenic response led to a sustained increase in vessel density, as demonstrated by a significantly greater number of CD31-positive vessels in the PD^@*L*2^ group versus all other groups at week 2. Consistent with the natural course of bone healing [30,31], vascularization peaked during the early chondral callus phase (week 2) and subsequently declined and underwent remodeling by the hard callus phase (week 4, Supplementary Fig. 6).

Residual implants can trigger inflammation and cause secondary injuries [32,33]. In order to further evaluate the safety of PD^@*L*2^, we measured the degradation kinetics of the material and cell persistence. The chiral fibrils were labeled with Thioflavin T (ThT, pseudo-colored green), which exhibits enhanced fluorescence intensity upon selective binding to *l*/*d*-GC18 nanofibrils (Supplementary Fig. 7). PDLSCs were labeled with the fluorescent dye CM-DiI (red) for cell membrane tracking (Supplementary Fig. 7). *In vivo* fluorescence imaging of rats with fresh bone defect and PD^@*L*2^ treatment revealed that the chiral fibrils were degraded within 24 h, while the CM-DiI signal from PDLSCs became barely detectable by 9 h (Supplementary Fig. 8). To further evaluate the biocompatibility of this system, we assessed its immunogenicity. Immunofluorescence analysis of implantation sites at days 3 and 7 revealed only transient, control-comparable macrophage (CD68^+^) infiltration that resolved by day 7, with no significant T lymphocyte (CD3^+^) accumulation, demonstrating the absence of a harmful adaptive immune response (Supplementary Fig. 9). Moreover, histopathology of major organs revealed no signs of inflammation or necrosis, indicating the systemic biocompatibility of the chiral fibrils (Supplementary Fig. 10).

Hence, we demonstrated that the PD^@*L*2^ can effectively induce osteogenesis and promote angiogenesis with good biocompatibility. Subsequently, further *in vitro* experiments were conducted to clarify the mechanisms behind the observed osteogenic–angiogenic coupling.

### 2.3 Helicity-dependent osteogenic differentiation of PDLSCs and resulting angiogenesis

**Fig. 3:**
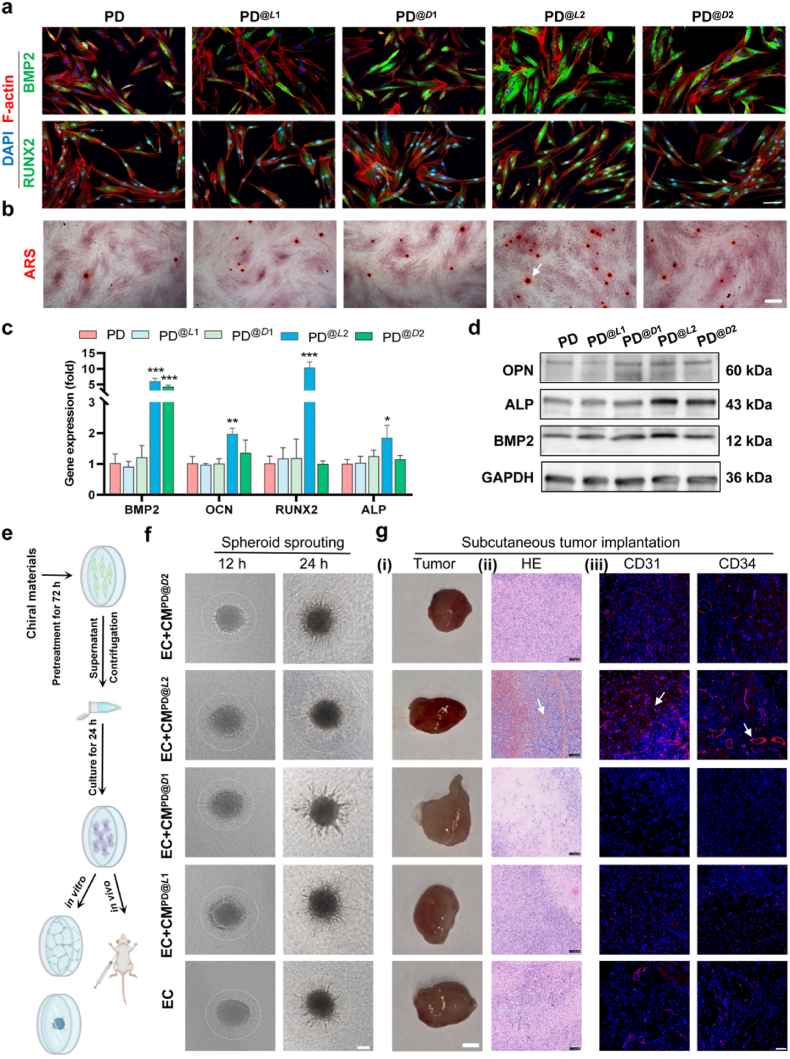
**Helicity-dependent osteogenic differentiation of PDLSCs and resulting angiogenesis. a**) Immunofluorescence localization of BMP2 and RUNX2 in PDLSCs after culture for 3 days in the chiral matrix (scale bar: 20 μm). b) Alizarin Red S (ARS) mineralization capacity staining of PDLSCs at day 21 (scale bars: 500 μm). c**)** RT-qPCR analysis of osteogenic marker expression (BMP2, OCN, RUNX2, ALP) in PDLSCs cultured for 3 days in a medium doped with chiral materials (∗*P* < 0.05, ∗∗*P* < 0.01, ∗∗∗*P* < 0.001 versus control; one-way ANOVA). d) Corresponding Western blot analysis of osteogenic marker protein expression. e) Experimental workflow for angiogenesis assays. f) Representative images of the HUVEC spheroid sprouting assay after treatment with different conditioned supernatants. White circles indicate the tips of sprouts (scale bars: 100 μm). g) In vivo vascularization analysis of xenograft tumors. From left to right: Photographs of resected tumors (scale bar: 3 mm). H&E staining of tumor sections reveals vascular structures with lumens. Immunofluorescence staining for CD31/CD34 (red) and cell nuclei with DAPI (blue); luminal structures are indicated by white arrows (scale bar: 50 μm).

To determine whether the observed *in vivo* osteogenesis originated from the helicity-dependent osteogenic differentiation of PDLSCs, we conducted a series of *in vitro* experiments. When PDLSCs were cultured with left-handed *L*2 fibrils, they exhibited robust osteogenic lineage commitment, as revealed by the significant upregulation of osteogenic markers (including osteocalcin (OCN), RUNX2, BMP2, and alkaline phosphatase (ALP)) compared to the control group (*P* < 0.05; Fig. 3a and Supplementary Fig. 12a). Remarkably, *L*2 fibrils resulted in a 10-fold increase of RUNX2 expression in PDLSCs compared to controls. The corresponding protein expression levels were detected by western blotting and immunofluorescence staining. It was observed that OPN, ALP, and BMP2 protein levels were significantly upregulated in *L*2-treated samples (Fig. 3b; *P* < 0.05, Supplementary Fig. 12b). Immunofluorescence staining revealed upregulated expression of BMP2 in the cytoplasm, while RUNX2, the master transcriptional regulator of osteogenesis, exhibited typical nuclear localization (Fig. 3c, Supplementary Fig. 12c). Moreover, the PD^@*L*2^ group displayed significantly increased ALP activity (Supplementary Fig. 13) and enhanced calcium nodule formation (Fig. 3d, Supplementary Fig. 12d).

We then sought to delineate the mechanisms by which left-handed nanofibrils promote angiogenesis. To this end, we performed a tube formation assay under three conditions [34]: (i) direct stimulation of HUVECs with the chiral fibrils, (ii) treatment of HUVECs with chiral fibril-stimulated PDLSC conditioned medium (CM), and (iii) direct co-culture of HUVECs with PDLSCs at a 4:1 ratio [35] (Supplementary Fig. 14). At the 4-h time point, direct chiral stimulation alone yielded limited pro-angiogenic effects. In contrast, the direct co-culture system demonstrated the most potent tube formation, thus suggesting synergy between integrated paracrine signaling and direct cell-contact mechanisms in initiating the process. By 8 h, however, the tube structures in the co-culture group regressed, whereas those in the CM group exhibited superior stability. This temporal shift underscores a key role for sustained paracrine signaling in maintaining the angiogenic network. Collectively, these results demonstrate that the pro-angiogenic effects of chiral nanofibrils are not direct but are primarily mediated by activating PDLSCs and enhancing their paracrine functions.

To validate this pro-angiogenic paracrine activity, we collected cell culture supernatants from PDLSCs pre-conditioned with left-handed *L*2 nanofibrils (designated CM^PD@*L*2^) and assessed their effects on HUVECs. The CM^PD@*L*2^ conditioned medium significantly promoted HUVEC viability, proliferation, and capillary formation compared to the control. Specifically, tube formation assays demonstrated that CM^PD@*L*2^ enhanced the formation of a markedly more complex and intricate tubular network, as evidenced by significant increases in junction numbers, tube length, and tube numbers relative to the untreated control group (*P* < 0.05, Supplementary Fig. 14). Consistent with this result, a sprouting assay revealed that CM^PD@*L*2^-treated HUVECs formed sprouts with significantly larger diameters (Fig. 3f; *P* < 0.05, Supplementary Fig. 12f). To substantiate these *in vitro* findings *in vivo*, we subcutaneously implanted HUVECs pretreated with conditioned medium (CM) into nude mice. After 4 days, the resulting tumors in the CM^PD@*L*2^ group displayed a distinct red color, indicating functional blood perfusion, in contrast to the pale control tumors (Fig. 3g(i)). Histological analysis revealed extensive angiogenesis. H&E staining showed pervasive microvessels with clear lumens in the CM^PD@*L*2^ group (Fig. 3g(ii)), and immunofluorescence staining revealed a significantly denser CD31+/CD34+ vascular network with increased lumen density (Fig. 3g(iii); *P* < 0.05, Supplementary Fig. 12e). Taken together, these data therefore demonstrate that the pro-angiogenic effects of *L*2 nanofibrils is mediated through potent paracrine activation of PDLSCs.

The robust angiogenesis induced by *L*2-treated PDLSCs is a principal factor in the observed bone regeneration. This aligns with the established role of vascular networks in tissue repair, which mediate nutrient delivery, waste clearance, and cell recruitment [36]. Hence, the enhanced paracrine activity of PDLSCs provides a mechanistic basis for the superior regenerative outcomes obtained with *L*2 treatment.

Our transcriptomic analyses at the 2-h timepoint confirmed that the left-handed *L*2 fibril fulfills its regenerative effect by rapid activation of both the osteogenic and angiogenic recruitment pathways in PDLSCs (Fig. 5a–b; Fig. 6b–c). Such an early, coordinated initiation is sufficient for triggering the essential pro-regenerative cascade *in vivo*, as affirmed by the observed successful bone regeneration, despite the 12-h retention of nanofibrils. These results are consistent with the reported clinical studies on the distribution of therapeutic regenerative cells *in vivo* [37,38], whereby stem cells modulate repair processes via paracrine signaling, even though they are quickly cleared after transplantation.

### 2.4 Helicity-dependent mechanotransduction pathway activation

To elucidate the underlying molecular mechanisms of the observed supramolecular helicity-dependent osteogenic and angiogenic crosstalk in PDLSCs, we performed RNA sequencing analysis on PDLSCs treated by *L*2 fibrils for 2 h to investigate the initial cellular response to matrix helicity.

**Fig. 4:**
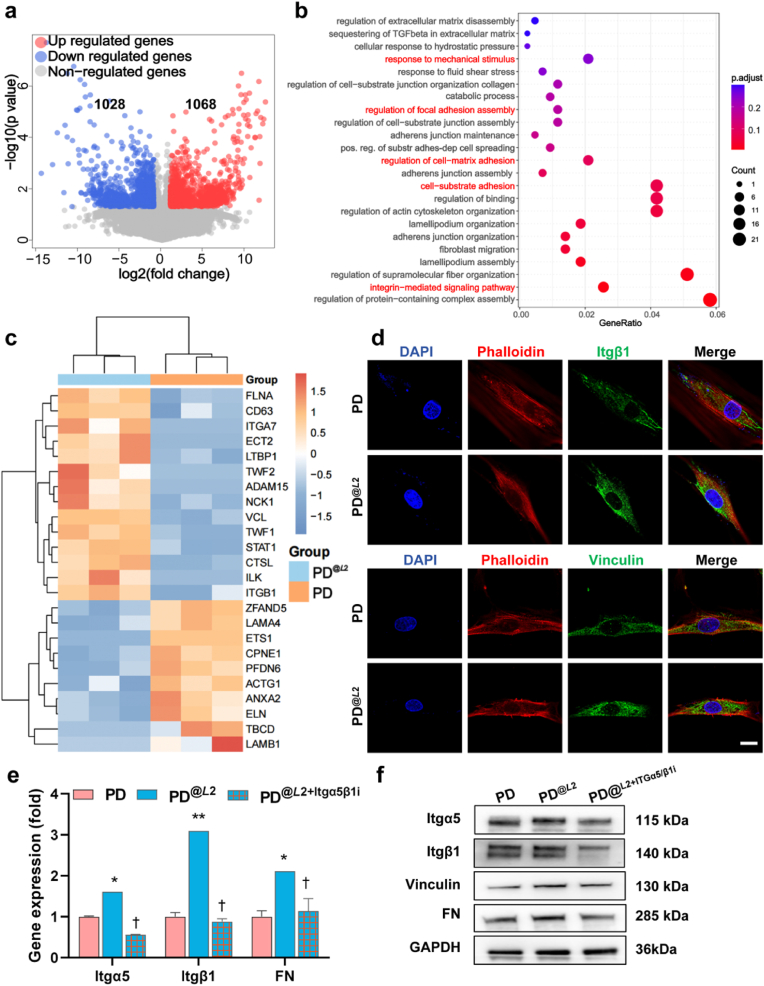
**Helicity-dependent mechano-transduction pathway activation.** a) Volcano plot showing differentially expressed genes. Red dots denote upregulated genes (log2FC > 1), blue dots denote downregulated genes (log2FC < −1), and gray dots represent non-regulated genes. b) Bubble plot of Gene Ontology Biological Process (GO-BP) enrichment analysis for the transcriptomic profiles of *L*2-treated PDLSCs. c) Heatmap showing the expression of genes related to cell adhesion, extracellular matrix (ECM), integrin signaling, and mechanotransduction. d) Immunofluorescence staining for detection of Itgβ1 and vinculin expression (DAPI: blue; scale bar: 10 μm). e) RT-qPCR quantification of Itgα5, Itgβ1, and FN mRNA levels, with or without 100 nM Cilengitide (Itgα5β1 inhibitor) (∗*P* < 0.05, ∗∗*P* < 0.01 vs. PD control; †*P* < 0.05 vs. PD^@*L*2^; one-way ANOVA). f) Western blot analysis of Itgα5, Itgβ1, vinculin, and FN protein expression levels, with or without Cilengitide treatment.

The volcano plots revealed significant differential regulation of gene expression in PDLSCs treated by *L*2 fibrils, with 1028 DEGs exhibiting upregulation and 1068 DEGs displaying downregulation (Fig. 4a). Pathway analysis of the transcriptional profiles using Gene Ontology (GO) revealed significant enrichment in pathways regulating cell adhesion and integrin-mediated signaling (Fig. 4b). Specifically, the upregulated transcripts were enriched in genes involved in cell-matrix adhesion (GO:0001952), integrin-mediated signaling pathway (GO:0007229), reaction to mechanical stimuli (GO:0009612), and regulation of actin cytoskeleton organization (GO:0032956). This pattern was reflected in the heatmap, which showed upregulation of multiple key genes related to cell adhesion and mechanotransduction (Fig. 4c). The upregulated genes included integrin beta-1 (ITGB1) and integrin-linked kinase (ILK), accompanied by elevated expression of vinculin (VCL), a critical focal adhesion protein [39]. Cytoskeletal regulatory genes, actin-binding proteins filamin A (FLNA) and twinfilin-1 (TWF1), along with microtubule-associated tubulin folding cofactor D (TBCD), demonstrated coordinated alterations [40,41]. Moreover, the activation of genes associated with fibronectin (FN)-dependent signaling pathways - including signal transducer and activator of transcription 1 (STAT1) - coupled with the downregulation of other extracellular matrix genes for recognition, including laminin subunit alpha-4 (LAMA4) and collagen type VIII alpha 1 chain (COL8A1), suggests that PDLSCs recognize *L*2 fibrils through FN [42].

**Fig. 5:**
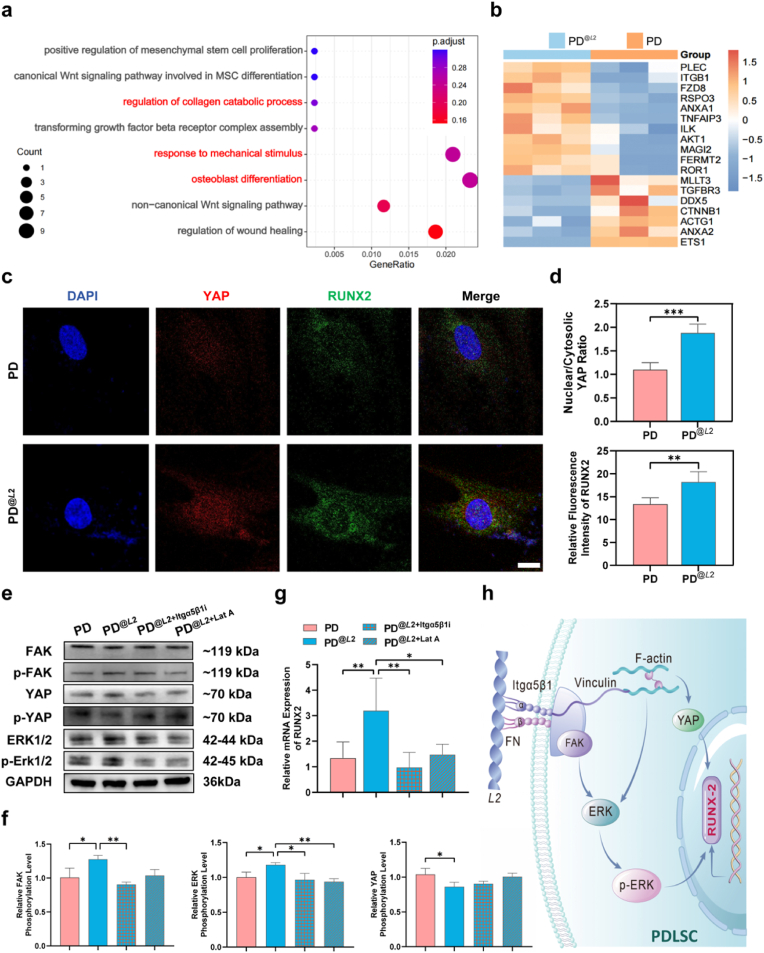
**Left-handed*****L*****2 fibrils promote osteogenesis via the FAK-ERK-YAP/RUNX2 mechanotransduction signaling axis.** a) Bubble plot of Gene Ontology Biological Process (GO-BP) enrichment analysis for osteogenic pathways in PDLSCs treated with *L*2 fibrils. b) Heatmap depicting the expression of osteogenic pathway-related genes. c) Representative immunofluorescence staining images showing the subcellular localization of YAP (red) and RUNX2 (green). *L*2 fibril treatment promotes nuclear translocation of YAP and enhances its co-localization with RUNX2. Scale bars: 10 μm. d) Quantitative analysis of the nuclear-to-cytoplasmic ratio of YAP and RUNX2. e) Western blot analysis showing *L*2 fibril-induced upregulation of p-ERK and p-YAP, which is reversed by treatment with Cilengitide, an inhibitor of integrin α5β1. f) Quantification of ERK, FAK and YAP phosphorylation levels from Western blot analyses. g) RT-qPCR analysis of RUNX2 expression in PDLSCs stimulated with *L*2 fibrils, with or without Cilengitide (integrin α5β1 inhibitor) or cytoskeletal inhibitor Latrunculin A (Lat A, 1 μM) pretreatment. Error bars indicate s.d.; ∗*P* < 0.05, ∗∗*P* < 0.01; one-way ANOVA. h) Schematic illustration of the proposed molecular signaling cascade that transduces the mechanosensitive potential of *L*2 fibrils to mediate osteogenic differentiation of PDLSCs.

**Fig. 6:**
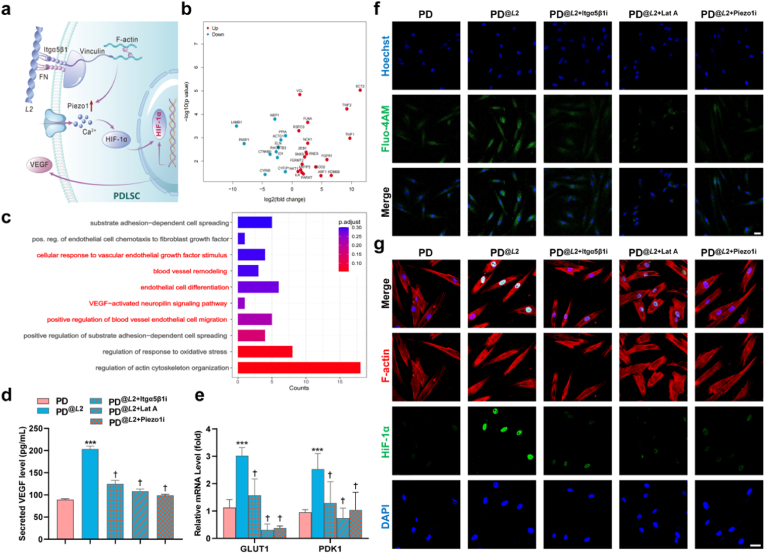
**Left-handed *L*2 fibrils promote angiogenesis via Piezo1–HIF-1α–VEGF paracrine signaling.** a) Schematic illustration of the Piezo1–HIF-1α–VEGF signaling axis, orchestrated by the mechanosensitive potential of *L*2 fibrils, which drives pro-angiogenic paracrine signaling in PDLSCs. b) Volcano plot showing differentially-expressed genes related to angiogenic pathways. c) Bar plot of Gene Ontology Biological Process (GO-BP) enrichment analysis for angiogenic pathways in PDLSCs treated with *L*2 fibrils. d) VEGF secretion quantified by ELISA. Data are presented as mean ± s.d.; ∗∗∗*P* < 0.001 vs. PD control; †*P* < 0.05 vs. PD^@*L*2^; one-way ANOVA. e) mRNA expression levels of HIF-1α-associated downstream genes GLUT1 and PDK1 under *L*2 stimulation. f) Live-cell Fluo-4 AM imaging illustrating *L*2-induced calcium influx, which is attenuated by inhibition of integrin α5β1 (Cilengitide), cytoskeletal inhibitor (Latrunculin A), or Piezo1 channel blockade (GsMTx4, 5 μM). Intracellular calcium was visualized using the fluorescent dye Fluo-4 AM (green), and cell nuclei were stained with Hoechst (blue). Scale bars: 50 μm. g) Representative immunofluorescence images of HIF-1α (green) and F-actin (red). Scale bars: 20 μm.

**Fig. 7:**
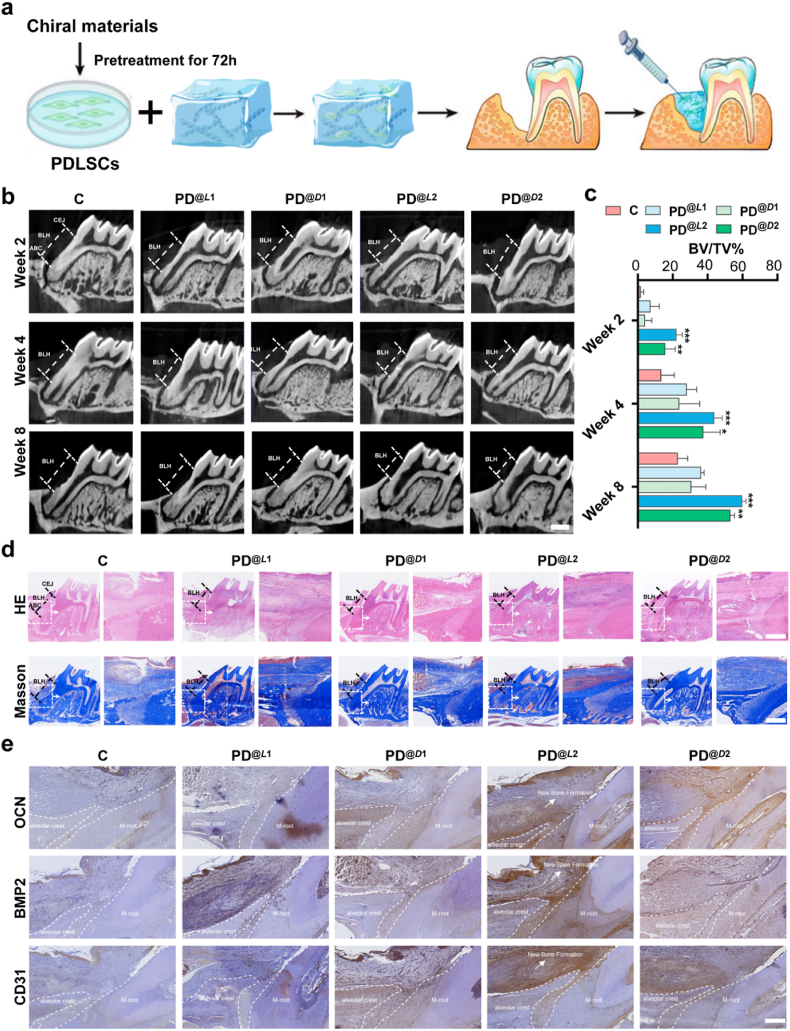
***L*2-PDLSC composites promote periodontal bone regeneration through osteo-vascular coupling.** a) Schematic illustration of the experimental procedure for implanting chiral fibrils pretreated PDLSCs following the creation of a maxillary groove-shaped defect at the mesial aspect of the first molar. (Defect dimensions: 0.5 × 0.5 × 1 mm^3^; *n* = 6/group; 6-week-old male Wistar rats) b) micro-CT reconstruction showing the temporal progression of bone regeneration at 2, 4, and 8 weeks post-implantation. Key anatomical landmarks, including alveolar bone crest (ABC), cemento-enamel junction (CEJ), and bone loss height (BLH), are indicated (scale bar: 500 μm). c) Quantitative assessment of osseous regeneration based on Micro-CT results showing dynamic changes in BV/TV% within the defect region (*n* = 6; error bars indicate s.d.; ∗*P* < 0.05, ∗∗*P* < 0.01, ∗∗∗*P* < 0.001 vs. control; one-way ANOVA). d) Histomorphometric evaluation: H&E and Masson's trichrome staining illustrating the microarchitecture of newly-formed bone tissues (scale bar: 200 μm). e) Molecular characterization: Immunohistochemical detection of osteogenic markers (OCN, BMP2) and vascular endothelial marker (CD31) expression patterns (scale bar: 100 μm).

To validate the pathways revealed by RNA sequencing analysis, we conducted RT-qPCR on selected gene markers. RT-qPCR analysis after 3-days of *L*2 treatment of PDLSCs showed significant activation of the FN-Itgα5β1-vinculin signaling axis (*P* < 0.05; Fig. 4e). This activation was further confirmed at the protein expression level. The Western blot results showed significant upregulation of Itgα5 and Itgβ1 in PDLSCs treated with *L*2 fibrils compared to the control group, which were inhibited by Cilengitide, an inhibitor of Itgα5β1 (Fig. 4f; *P* < 0.05, Supplementary Fig. 15). Furthermore, immunofluorescence staining revealed that treatment with *L*2 fibrils promoted the clustering and upregulation of Itgα5β1 in PDLSCs compared to other chiral structures (Fig. 4d; Supplementary Fig. 16). The observed clustering and upregulation of Itgα5β1 is a key event in cell adhesion. Focal adhesions are assembled by integrin receptors and adaptor proteins, such as vinculin [43], which in turn transduce bidirectional (outside-in/inside-out) signals to regulate adhesion, migration, and differentiation [44]. Our transcriptomic data, corroborated by other molecular analyses, elucidated that *L*2 fibrils initiate mechanotransduction through Itgα5β1-FN clustering.

### 2.5 Left-handed *L*2 fibrils promote osteogenesis via the FAK-ERK-YAP/RUNX2 mechanotransduction signaling axis

We next investigated how *L*2 fibrils activate intracellular signaling through integrin-mediated mechanotransduction to promote osteogenic differentiation in PDLSCs. Transcriptome analysis revealed that a 2-h treatment with *L*2 fibrils significantly upregulated early osteogenic pathways, including osteoblast differentiation (GO:0001649) and regulation of collagen catabolic processes (GO:0010710) (Fig. 5a). Consistent with these findings, key genes markers associated with nuclear activation were significantly altered in *L*2-treated PDLSCs, such as R-spondin 3 (RSPO3), frizzled class receptor 8 (FZD8), and Annexin A1 (ANXA1) (Fig. 5b).

Yes-associated protein (YAP), a major downstream effector of the Hippo pathway, partners with TEAD transcription factors to activate mechanotransduction signaling and promote osteogenic differentiation of stem cells [45]. In this study, Western blot analysis revealed that *L*2 treatment significantly reduced the p-YAP (Ser 127)/Total YAP ratio, thereby establishing the biochemical basis for YAP's transition from an inhibited to an activated state (Fig. 5f). Consistent with this finding, immunofluorescence staining showed that *L*2 fibrils promoted the nuclear translocation of YAP and RUNX2 (Fig. 5c), thereby initiating early osteogenic differentiation of PDLSCs. Quantitative analysis further confirmed that *L*2 treatment led to a significant increase in both the nuclear-to-cytoplasmic ratio of YAP and the nuclear accumulation of RUNX2 compared with the control group (*P* < 0.01, Fig. 5d). These results thus suggest that *L*2 fibrils amplify mechanosensing in PDLSCs and promote early nuclear entry of YAP, thereby initiating the expression of upstream osteogenic markers such as RUNX2.

To elucidate how *L*2 fibrils transduce mechanical cues into pro-osteogenic signaling, we assessed the role of the integrin-cytoskeleton-YAP axis. Notably, the L2-induced transcriptional upregulation of RUNX2 was reversed by treatment with Cilengitide (an integrin α5β1 inhibitor) or the cytoskeletal inhibitor latrunculin A (*P* < 0.05, Fig. 5g). This result indicates that integrin binding and cytoskeletal integrity are essential for initiating and transmitting the mechanical signals driven by *L*2, which is consistent with the established mechanism whereby cytoskeletal tension facilitates YAP nuclear import by enlarging nuclear pores, thereby potentiating osteogenesis [46].

Integrin-mediated mechanotransduction typically involves activation of focal adhesion kinase (FAK) and extracellular signal-regulated kinase (ERK1/2) pathways [47,48]. Western blot analysis of *L*2-treated PDLSCs revealed activation of these pathways, showing increased phosphorylation of ERK1/2 and FAK, along with elevated RUNX2 expression (*P* < 0.05, Fig. 5e and f). These effects were attenuated by Cilengitide, which confirmed dependency on integrin α5β1. Correspondingly, a significant reduction in ALP-positive cells (day 7) and subsequent mineralized nodule formation (day 21) was observed in groups treated with Cilengitide or Latrunculin A, as compared to the *L*2-treated controls (*P* < 0.01; Supplementary Fig. 17).

Based on these results, we hypothesize that the left-handed *L*2 fibrils create a pro-osteogenic microenvironment that enhances PDLSC differentiation by amplifying integrin α5β1-mediated mechanosensing and downstream mechanotransduction. The key events involve integrin binding, cytoskeletal force transmission, FAK/ERK signaling activation, and nuclear translocation of YAP/RUNX2 (Fig. 5h).

### 2.6 Left-handed *L*2 fibrils stimulate angiogenesis via the piezo1-HIF-1α-VEGF paracrine signaling axis

Based on *in vitro* evidence of a pro-angiogenic paracrine mechanism in *L*2-treated PDLSCs, we further investigated how the physical cues presented by *L*2 fibrils are transduced into intracellular chemical signals that drive this paracrine response. Transcriptomic profiling after 2-h treatment with left-handed *L*2 fibrils revealed significant upregulation of key angiogenic pathways, including positive upregulation of endothelial cell migration (GO:0045446), blood vessel remodeling (GO:0001974), and VEGF-activated neuropilin signaling (GO:0038190) (Fig. 6b). A volcano plot further highlighted differentially-expressed genes involved in angiogenic activation, such as Epithelial Cell Transforming Sequence 2 (ECT2), Twinfilin-2 (TWF2), and Filamin A (FLNA) (Fig. 6c).

ELISA quantification confirmed that *L*2 stimulation significantly enhanced VEGF secretion by PDLSCs to 203 ± 6.47 pg/mL—a 2.3-fold increase over the control (89.23 ± 2.15 pg/mL, *P* < 0.01; Fig. 6d). VEGF is a well-established pro-angiogenic growth factor that also supports osteogenic differentiation [49,50], positioning it as a central effector in the *L*2-induced paracrine cascade.

Piezo1, a mechanosensitive Ca^2+^channel, is an important transducer of biomechanical signals in tissue homeostasis and repair [51], including its reported involvement in tensile stress-induced osteogenic [52,53] and endothelial differentiation [54] in periodontal ligament cells. We therefore investigated the role of Piezo1 in L2-mediated pro-angiogenic signaling. Using Fluo-4 AM calcium imaging, we observed a pronounced increase in intracellular Ca^2+^levels in *L*2-stimulated PDLSCs within 30 min. This response was significantly suppressed by the Piezo1 inhibitor GsMTx4, the integrin α5β1 inhibitor Cilengitide, as well as the cytoskeletal disruptor Latrunculin A (Fig. 6f; *P* < 0.05, Supplementary Fig. 18b), thus indicating that integrin engagement, Piezo1 activation, and cytoskeletal integrity are all required for *L*2-evoked Ca^2+^ influx.

Immunofluorescence staining further showed that *L*2 fibrils promote HIF-1α stabilization and nuclear accumulation. This effect was inhibited by cytoskeletal disruption, which was induced by Latrunculin A and was manifested as a loss of the characteristic spindle-like morphology and an increase in cell rounding (Fig. 6g). The functional impact of HIF-1α activation was confirmed by the transcriptional upregulation of its canonical targets, GLUT1 (Glucose Transporter 1) and PDK1 (Pyruvate Dehydrogenase Kinase 1), key drivers of metabolic reprogramming [55] (*P* < 0.01, Fig. 6e). Importantly, inhibition of upstream components—integrin α5β1, the cytoskeleton, or the mechanosensor Piezo1—abolished this HIF1α-mediated response, while concurrently attenuating VEGF secretion and suppressing both tube formation and spheroid sprouting (*P* < 0.05, Supplementary Figs. 18a and 19).

Collectively, these findings support a model in which left-handed *L*2 fibrils activate an integrin-mediated mechanotransduction signaling pathway that is transmitted via the cytoskeleton to stimulate Piezo1-channel activity, triggering Ca^2+^influx and HIF1α-dependent transcription, ultimately enhancing VEGF secretion and paracrine angiogenesis (Fig. 6a).

### 2.7 Therapeutic application of L2-PDLSCs for periodontal bone regeneration

Alveolar bone defect is a prevalent clinical condition that leads to impaired oral function and aesthetics [56], causing physical and psychological distress, and its reconstruction poses a significant clinical challenge [57] Periodontal regeneration aims to fully restore both the architecture and biomechanical function of periodontal tissues following inflammation resolution [58]. Based on the observed potency of left-handed *L*2 fibrils in inducing the osteogenic differentiation of PDLSCs, we sought to evaluate their therapeutic potential for repairing alveolar bone defects.

Composites of chiral material and PDLSCs were implanted into surgically created alveolar bone defects (0.5 × 0.5 × 1 mm^3^) within the mesial region of the maxillary first molars in Wistar rats, whereas the control group received surgery without any implanted cells. Tissue specimens were collected at 2, 4, and 8 weeks post-operation to evaluate osteogenic and angiogenic outcomes within the defect regions (Fig. 7a).

Micro-CT analysis revealed significantly reduced bone loss height (BLH)—measured from the mesial alveolar bone crest (ABC) of the defect to the cemento-enamel junction (CEJ) [59]—in the PD^@*L*2^ group, as compared to that in the control group at all time points (2, 4, and 8 weeks), which demonstrated enhanced periodontal bone regeneration within the alveolar bone defects (Fig. 7b and c; Supplementary Fig. 20). The PD^@*L*2^ group exhibited a progressive increase in bone volume fraction (BV/TV), with values of 22.19 ± 3.4 % at 2 weeks, 43.89 ± 4.54 % at 4 weeks, and 59.8 ± 2.42 % at 8 weeks, which were significantly higher than those in the control group (1.66 ± 1.62 %, 13.4 ± 7.51 %, and 22.94 ± 5.62 %, respectively; Fig. 7c). These results underscore the potent osteogenic effects of left-handed *L*2 fibrils. Histological staining showed densely arranged neo-bone tissue, accompanied by increased osteoblast activity and well-defined trabecular structures in the PD^@*L*2^ group, whereas the defects in the control group exhibited sparse and disorganized new bone formation (Fig. 7d). Furthermore, immunohistochemical staining confirmed markedly upregulated expression levels of osteogenic markers (BMP2, OCN) together with the angiogenesis-related marker CD31 in the PD^@*L*2^-treated defects, as compared to the controls (Fig. 7e).

The cumulative evidence thus indicates that *L*2 facilitates the repair of alveolar bone defects. As adult stem cells derived from the neural crest [60], PDLSCs secrete VEGF during osteogenic differentiation, which recruits vascular endothelial cells and potentiates vasculogenesis in engineered tissues. PDLSCs possess pericyte-like characteristics and are localized adjacent to endothelial cells, which contribute to the stability of the capillary-like structure *in vivo* [61,62]. The *L*2-PDLSC composites thus provide not only a promising therapeutic strategy but also a conceptual framework for clinical periodontal regeneration.

## 3 Conclusion

In conclusion, we synthesized supramolecular fibrils via the co-assembly of chiral amino acid derivative enantiomers (*l*/*d*-GC18) and a bridging pyrazine molecule. The helicity of the fibrils depended on both the molecular chirality of the amino acid and the stoichiometric ratio of the two components. Both *in vivo* and *in vitro* studies collectively showed that molecular chirality and supramolecular helicity can act synergistically. The left-handed fibrils assembled from *l*-GC18 and pyrazine promoted the osteogenic differentiation of periodontal ligament stem cells (PDLSCs), which accelerated bone formation in both calvarial and alveolar bone defect models. The regeneration rate exceeded that achieved by bone marrow mesenchymal stem cells (BMSCs) in previous studies. Sequencing and molecular biology experiments jointly revealed that the osteogenic-angiogenic coupling mechanism facilitates the helicity-dependent bone regeneration of PDLSCs. Based on these findings, we propose a promising therapeutic strategy for tissue regeneration that is not only effective but also addresses ethical and safety concerns.

However, this dental-derived, stem-cell-dependent osteogenic strategy awaits further validation in future clinical trials before widespread clinical application. Moreover, a compelling frontier lies in exploring the chirality-dependent fate determination of dental stem cells for diverse regenerative scenarios, such as neural repair. Realizing this vision—and redefining dental tissues from surgical waste into a versatile therapeutic pillar—hinges on the development of next-generation chiral matrix materials and a fundamental dissection of the underlying mechanisms.

## CRediT authorship contribution statement

**Zhuohang Deng:** Writing – original draft, Methodology. **Meijun Li:** Writing – review & editing, Methodology, Conceptualization. **Yifan Wang:** Project administration, Formal analysis, Data curation. **Wenjing Li:** Supervision. **Zijian Gong:** Software. **Peiwen Liao:** Visualization. **Shengzhen Luo:** Methodology. **Minghua Liu:** Writing – review & editing. **Xuliang Deng:** Writing – review & editing, Resources, Funding acquisition.

## Ethics approval and consent to participate

All animal experiments were conducted with the approval of the Animal Care and Use Committee of Peking University (IACUC number: BDKQ-202502250521).

## Declaration of competing interest

All authors declare that there are no competing interests.
