# Supplementary material for: Supramolecular helicity dependent osteogenesis and angiogenesis crosstalk of periodontal ligament stem cell
Source: Bioact Mater. 2026 Jan 2;59:450–62. doi: 10.1016/j.bioactmat.2025.12.057 (PMC12805306; doi:10.1016/j.bioactmat.2025.12.057)
Supplement: Multimedia component 1 [file mmc1.docx]

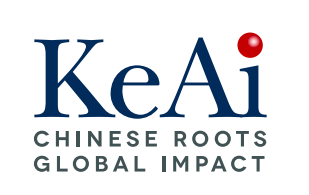


Supporting Information

**Supramolecular Helicity Dependent Osteogenesis and Angiogenesis Crosstalk of Periodontal Ligament Stem Cell**

**Supramolecular Helicity Dependent Osteogenesis and Angiogenesis Crosstalk of Periodontal Ligament Stem Cell**

**Table of Contents**

[1. Chiral materials testing instruments 3](#_Toc173666929)

[2. Synthesis and characterization](#_Toc173666930) 3

[3. Materials and methods in vivo](#_Toc173666935) 9

[4. Expression of osteogenic and angiogenic markers in vivo](#_Toc173666936) 11

[5. In vivo degradation and cell retention](#_Toc173666936) 12

[6. Biosafety validation in vivo](#_Toc173666936) 14

[7. Materials and methods in vitro](#_Toc173666931) 15

[8. Biocompatibility assessment of PDLSCs and HUVECs](#_Toc173666932) 18

[9. Quantitative Analysis of osteogenesis and angiogenesis 1](#_Toc173666933)9

[10. ALP Staining](#_Toc173666936) 19

[11. Chiral material-mediated angiogenesis in HUVECs](#_Toc173666935) 20

[12. Chiral matrix-selective integrin activation 21](#_Toc173666934)

[13. Inhibition of the Itgα5β1-cytoskeleton axis reverses](#_Toc173666936) *[L](#_Toc173666936)*[2-driven osteogenesis](#_Toc173666936) 23

[14. Reversal of](#_Toc173666936) *[L](#_Toc173666936)*[2-driven angiogenesis by Inhibiting the Itgα5β1-cytoskeleton-Piezo1 axis](#_Toc173666936) 24

[15. Micro-CT quantification of defect bone height](#_Toc173666936) 26

[16. Primers of the genes used in RT-qPCR](#_Toc173666936) 27

[References:](#_Toc173666937) 28

# Chiral materials testing instruments

Atomic Force Microscopy (AFM): Samples were deposited onto freshly cleaved mica substrates, air-dried, and subsequently imaged using a Bruker Multimode 8 AFM system operating in tapping mode (scan rate: 1 Hz, resolution: 512 × 512 pixels).

Cryo-Electron Microscopy (Cryo-EM): Samples were vitrified on Quantifoil R1.2/1.3 grids (300 mesh) and imaged under low-dose conditions (<20 e⁻/Å²) using a JEOL JEM-2100F TEM equipped with a field-emission gun (accelerating voltage: 200 kV, magnification: 50,000×).

Circular Dichroism (CD): Spectra were recorded from 190 to 350 nm (bandwidth: 1 nm, data pitch: 0.5 nm) on a JASCO J-1500 spectropolarimeter equipped with a temperature-controlled cuvette holder (25 ± 0.1°C, path length: 1 mm).

Fourier Transform Infrared Spectroscopy (FTIR): Samples were cast onto calcium fluoride windows (10 × 10 × 0.5 mm) and analyzed using a Nicolet iS50 FTIR spectrometer (resolution: 4 cm⁻¹, 32 scans) under a continuous nitrogen purge.

X-ray Diffraction (XRD): Patterns were collected on a Bruker D8 Advance diffractometer (Cu-Kα radiation, λ = 1.5418 Å) over a 2θ range of 5-50° with a step size of 0.02° using sample-loaded glass slides.

# Synthesis and characterization

# *l*-GC

Step 1:

Step 2:

***d*-GC**

Step 1:

Step 2:

**Supplementary Scheme 1.** Synthesis routes to *l*-GC and *d*-GC.

**Compound *l/d*-GC**

Diethyl glutamate hydrochloride (7.2 g, 1.0 equiv) was suspended in 150 mL of dichloromethane (DCM), followed by the addition of triethylamine (9 mL). After stirring for 0.5 h, stearic acid (8.55 g, 1.0 equiv) was added under ice bath cooling. Subsequently, HOBt (4.8 g, 1.2 equiv) and EDCI (6.9 g, 1.2 equiv) were added to the reaction mixture. After 3 h, the ice bath was removed, and the reaction was allowed to proceed at room temperature for 7 days. The solvent was evaporated under reduced pressure, and the crude product was recrystallized twice from ethanol.

The primary product was dispersed in 100 mL of mixed solvent (EtOH/THF, 1:1 v/v), followed by addition of 50 mL of aqueous LiOH solution (5 g, excess). The reaction mixture was stirred at room temperature for 7 days. After solvent removal under reduced pressure, the crude product was purified by double recrystallization from ethanol.

**Assembly conditions**

CRC molecules were dispersed in ultrapure water (5 mg/mL), followed by the addition of a pre-prepared pyrazine stock solution to achieve a CRC-to-pyrazine molar ratio of 1:2. The mixture was then heated to boiling with simultaneous ultrasonication in a water bath until the system became nearly transparent. After cooling to room temperature, a white colloidal solution was obtained, which was subsequently allowed to assemble for an additional 3 days under static conditions at room temperature. At a 1:1 molar ratio (*l*/*d*-GC:Pz), the system formed non-helical *L1* (*l*-form) and *D1* (*d*-form) fibrils. At a 1:2 molar ratio (*l*/*d*-GC:Pz), the assemblies developed helical architectures (*L*2 and *D*2 respectively)

**Morphological characterization**

Atomic force microscopy (AFM) and Cryo-Electron Microscopy (Cryo-EM) characterization revealed that the chiral self-assembly behavior was precisely modulated by both molecular chirality and stoichiometric ratios. Specifically, at a 1:1 ratio of *l*-GC to Pz, non-helical *L*1 (*l*-form) fibrils formed, exhibiting chirality only at the molecular level. In contrast, a 1:2 ratio of *l*-GC to Pz resulted in left-handed helical *L*2 architectures that displayed both molecular chirality and emergent supramolecular helicity. Similarly, a 1:1 ratio of *d*-GC to Pz led to non-helical *D*1 (*d*-form) fibrils with molecular-level chirality, while a 1:2 ratio produced right-handed helical *D*2 superstructures demonstrating chirality across both molecular and supramolecular levels. This stoichiometry-controlled hierarchical chirality transition from molecular to supramolecular level is particularly significant as the helical *L*2/*D*2 assemblies successfully mimic key extracellular matrix (ECM) features by providing chiral recognition interfaces and helical topological cues, thereby establishing biomimetic scaffolds for subsequent biological investigations and offering new possibilities for designing biofunctional chiral nanomaterials.

#
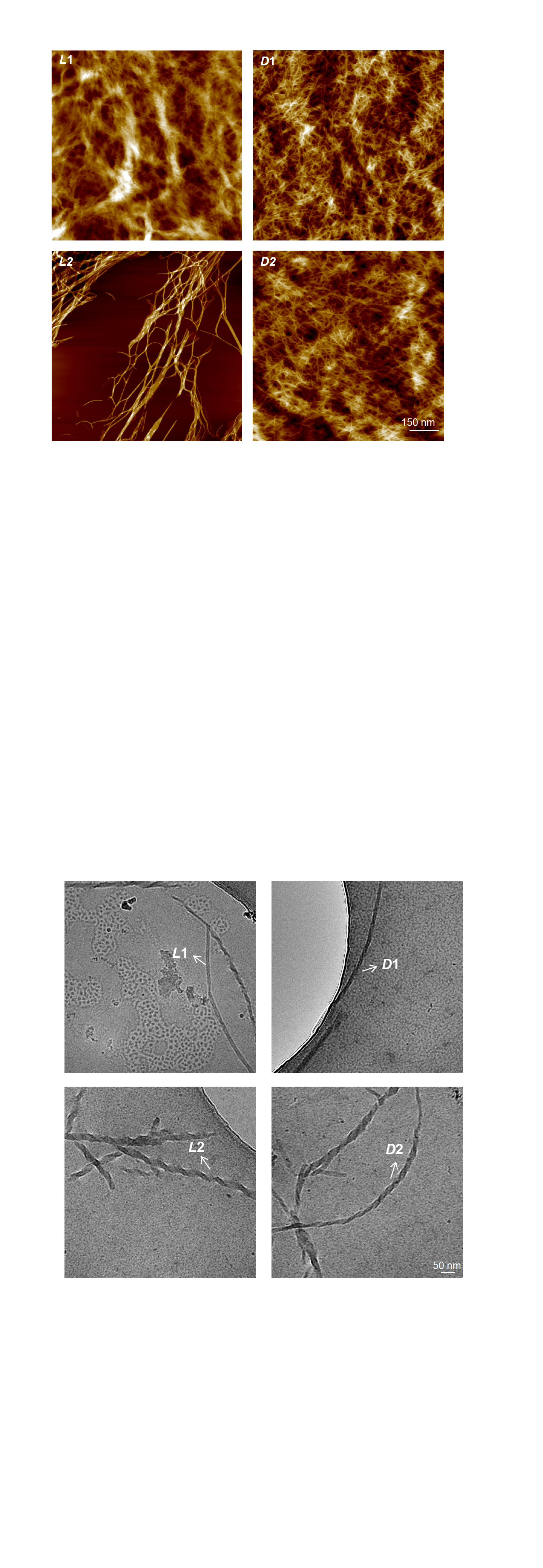


# **Supplementary Figure 1**.**** Atomic force microscopy (AFM) topographic images.

#
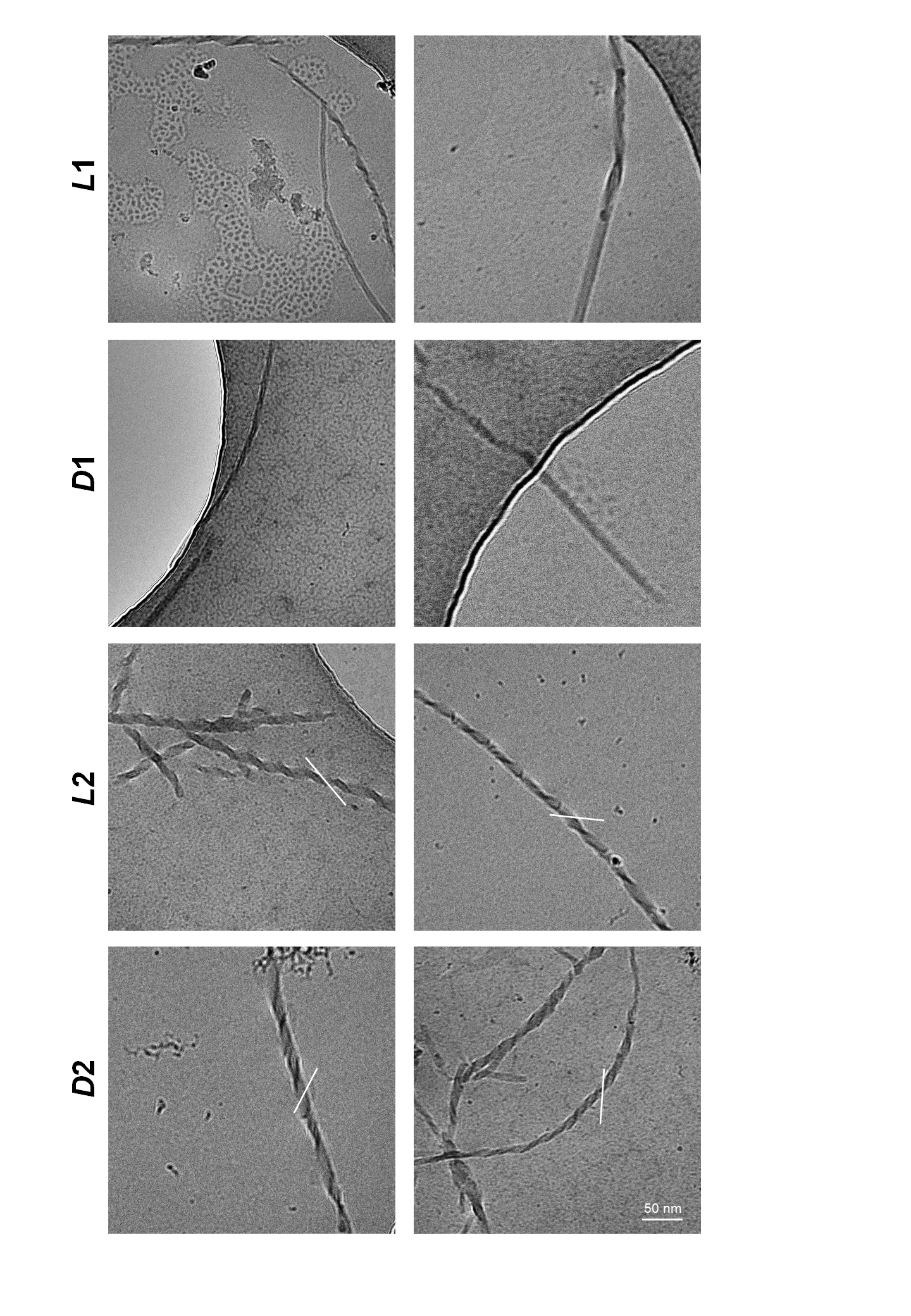


# ****S**upplementary Figure 2**.**** Cryo-EM visualization of chiral nanostructures (scale bar: 50 nm).

#
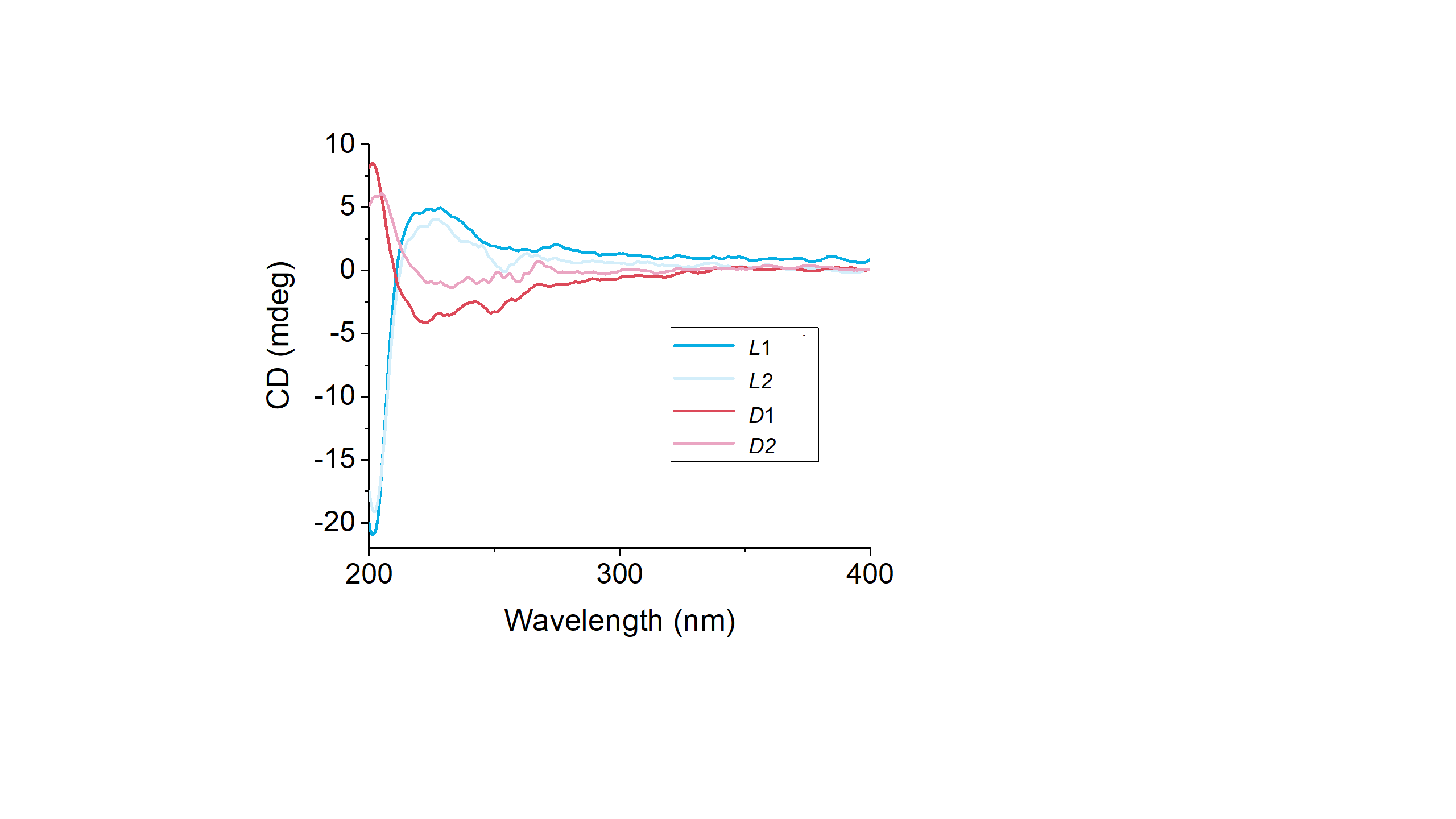


# ****S**upplementary Figure 3**. Circular dichroism (CD) spectra demonstrating distinct chiral signatures.


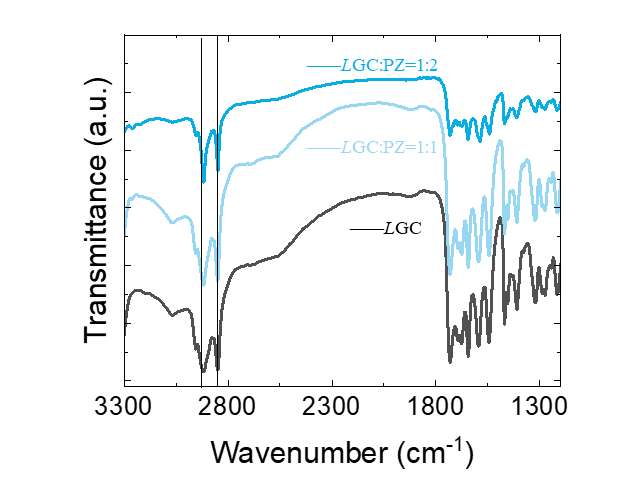


# ****S****upplementary Figure 4. FTIR spectra of chiral nanostructures.

# Materials and methods *in vivo*

**Animal Models**

The experimental protocol was approved by the Animal Care and Use Committee of Peking University (BDKQ-202502250521).

Calvarial Critical-Sized Defect Model ^1^: To establish clinical relevance, male Sprague-Dawley rats (6-week-old, SPF-grade) were subjected to bilateral 5-mm full-thickness calvarial defects under sodium pentobarbital anesthesia (40 mg/kg, i.p.). PDLSCs were stimulated for 3 days with complete medium containing 20 μg/mL of chiral materials (four experimental groups: *L*1, *D*1, *L*2, *D*2). Following harvesting, the cells were gently mixed with the chiral fibril sol by pipetting up and down 10-15 times to form a homogeneous cell-laden sol. This composite exhibited suitable viscosity for adhering to the bone defect site. The animals were randomized into five cohorts (n=6/group): four treatment groups receiving PDLSC-seeded chiral fibrils (PDLSCs@*L*1/*D*1/*L*2/*D*2, 5 × 10⁵ cells/implant) and one untreated control. All surgical sites were protected with resorbable collagen membranes (Bio-Gide®, 0.2 mm thickness) to prevent soft tissue invasion. Longitudinal assessments were conducted as follows: First, the degradation of ThT-labeled chiral fibrils and the retention of CM-DiI-labeled PDLSCs were monitored by *in vivo* imaging within the first 48 hours post-operation. Subsequently, tissue samples from the defect sites were harvested on days 3 and 7 for immunofluorescence staining to assess early immune responses. Angiogenesis was evaluated using optical coherence tomography angiography (OCTA) at the 2-week timepoint. Finally, terminal analyses were performed at 4, 8, and 12 weeks, which included micro-computed tomography (micro-CT) for bone morphometry and histomorphometric characterization using hematoxylin and eosin (H&E), Masson's trichrome, immunofluorescence, and immunohistochemical staining.

Subcutaneous Tumorigenesis ^2^: Eight-week-old male BALB/c nude mice (SPF-grade) were randomly allocated into five experimental groups (*n*=5/group): EC + CM^PD^, EC + CM^PD@^*^L^*^1^, EC + CM^PD@^*^D^*^1^, EC + CM^PD@^*^L^*^2^ and EC + CM^PD@^*^D^*^2^. Following 72 hours of pretreatment with chiral materials (*L*1/*D*1/*L*2/*D*2), PDLSC-conditioned media were collected and used to culture HUVECs for 24 hours. The treated HUVECs (1 × 10⁸ cells/mL) were then mixed 1:1 with growth factor-reduced Matrigel (Corning) and 200 μL aliquots (containing 2 × 10⁷ cells) were subcutaneously injected into the dorsal region. On day 4 post-surgery, the implants were excised, fixed in 4% (w/v) paraformaldehyde for 24 hours, and processed for paraffin embedding. Histological evaluation included H&E staining and immunofluorescence microscopy.

Alveolar Bone Defect Model ^3^: Standardized three-walled bone defects (0.5 mm mesiodistal width × 0.5 mm buccolingual width × 1 mm depth) were created in the mesial alveolar bone of the maxillary first molars in six-week-old male Wistar rats (*n* = 6 per group) to validate site-specific regeneration. All surgical procedures were performed under aseptic conditions using a low-speed handpiece (Piezosurgery®) operated at controlled speeds with a diamond bur. Animals were randomly allocated to five experimental groups: four treatment groups receiving chiral fibril-pretreated PDLSCs (PD^@^*^L^*^1^, PD^@^*^D^*^1^, PD^@^*^L^*^2^, PD^@^*^D^*^2^, 5 × 10⁵ cells/implant) and one sham-operated control. The defect sites were implanted with chiral matrix-PDLSCs constructs, covered with resorbable collagen membranes (Bio-Gide®, 0.2 mm thickness), and sutured using interrupted 6-0 polypropylene. Postoperative evaluation at 2, 4, and 8 weeks included: (i) quantitative micro-CT analysis of bone volume fraction (BV/TV), (ii) histomorphometric assessment via Goldner's trichrome and Masson's staining, and (iii) immunofluorescence quantification of osteogenic markers (RUNX2 and OCN).

**In Vivo Testing**

In Vivo Imaging: The chiral fibrils were fluorescently labeled with Thioflavin T (ThT) at a concentration of 1 mg/mL. Periodontal ligament stem cells (PDLSCs) were stained with the lipophilic dye CM-DiI (Beyotime, C1991S-3) according to the manufacturer's instructions to label the cell membranes. The following experimental groups were established for *in vivo* tracking: Blank, PBS control with ThT (PBS^ThT^), chiral fibrils with ThT (*L*2^ThT^), PDLSCs with DiI (PDLSCs^DiI^), and the composite of chiral fibrils and PDLSCs (*L*2^ThT^ + PDLSCs^DiI^). Post-implantation, fluorescence signals were acquired hourly using a small animal *in vivo* imaging system. The ThT signal was detected at an excitation/emission of 420/570 nm, and the DiI signal at 560/620 nm. The fluorescence intensity of the region of interest (ROI) was quantified for each time point to generate the degradation curve of the chiral fibrils and the persistence curve of the PDLSCs.

Optical Coherence Tomography Angiography (OCTA): Optical coherence tomography angiography (OCTA) was employed to quantitatively assess angiogenesis parameters, including bone density, vascular area density, and lumen diameter, using the AngioVue mode with an axial resolution of 5 μm.

Micro-Computed Tomography (Micro-CT): (i) Cranial Bone Defect Analysis: The newly formed bone tissue in the cranial defect area was quantitatively analyzed using a micro-computed tomography (micro-CT) system (Scanco Medical, Bassersdorf, Switzerland). The raw scan data were reconstructed into three-dimensional models using CTvox software (SkyScan, Bruker), and the bone volume fraction (BV/TV) was calculated with CTAn analysis software. (ii) Periodontal Bone Defect Analysis: In the periodontal bone defect model, the maxillary molars were examined from buccal, palatal, and occlusal views to identify the cemento-enamel junction (CEJ) and the alveolar bone crest (ABC). On two-dimensional coronal sections, the slice showing the lowest point of the mesial bone defect was selected for further analysis. Serial sectioning was performed to identify a three-wall bone defect measuring 0.5 mm in mesiodistal width, 0.5 mm in buccolingual width, and 1 mm in depth. Bone volume/total volume (BV/TV) was subsequently calculated to assess bone density within the defect region. Additionally, the bone loss height (BLH) was quantitatively measured as the distance from the mesial ABC of the defect to the CEJ.

Hematoxylin and Eosin (H&E) Staining Analysis: Conventional hematoxylin and eosin staining was performed on 5μm decalcified cranial bone tissue sections. Following nuclear staining with hematoxylin and cytoplasmic staining with eosin, the morphological structure of the newly formed bone tissue, inflammatory cell infiltration, and material degradation in the defect area were observed under an optical microscope.

Masson's Trichrome Staining Analysis: Masson's trichrome staining was conducted using a three-step process, which included Weigert's iron hematoxylin nuclear staining, picrosirius red-fuchsin cytoplasmic staining, and aniline blue collagen fiber staining. This method enabled clear differentiation between cellular components and collagen fiber distribution within the bone tissue. The collagen deposition area percentage in the defect region was quantitatively analyzed using image analysis software to evaluate the degree of fibrosis during bone repair.

Immunofluorescence Staining Analysis: Following antigen retrieval, the sections were incubated overnight at 4°C with the following primary antibodies from Abcam: anti-CD3 (ab16669), anti-CD68 (ab283654), anti-BMP2 (ab284387), anti-RUNX2 (ab192256), and anti-OPN (ab283669). Subsequently, appropriate fluorescence-labeled secondary antibodies were applied and incubated in the dark. Cell nuclei were counterstained with DAPI. The expression of osteogenic markers and angiogenesis-related structures was examined and quantitatively analyzed using a laser confocal microscope, providing insights into the characteristics of the bone regeneration microenvironment.

Immunohistochemical Staining Analysis: A standard protocol involving peroxidase blocking, antigen retrieval, and serum blocking was employed. The sections were then incubated overnight at 4°C with the following primary antibodies from Abcam: anti-OCN (HUABIO, HA723601), anti-BMP2 (ab284387), and anti-CD31 (HUABIO, ER31219). Immunostaining was subsequently performed using appropriate horseradish peroxidase (HRP)-conjugated secondary antibodies (Servicebio).

# Expression of osteogenic and angiogenic markers *in vivo*


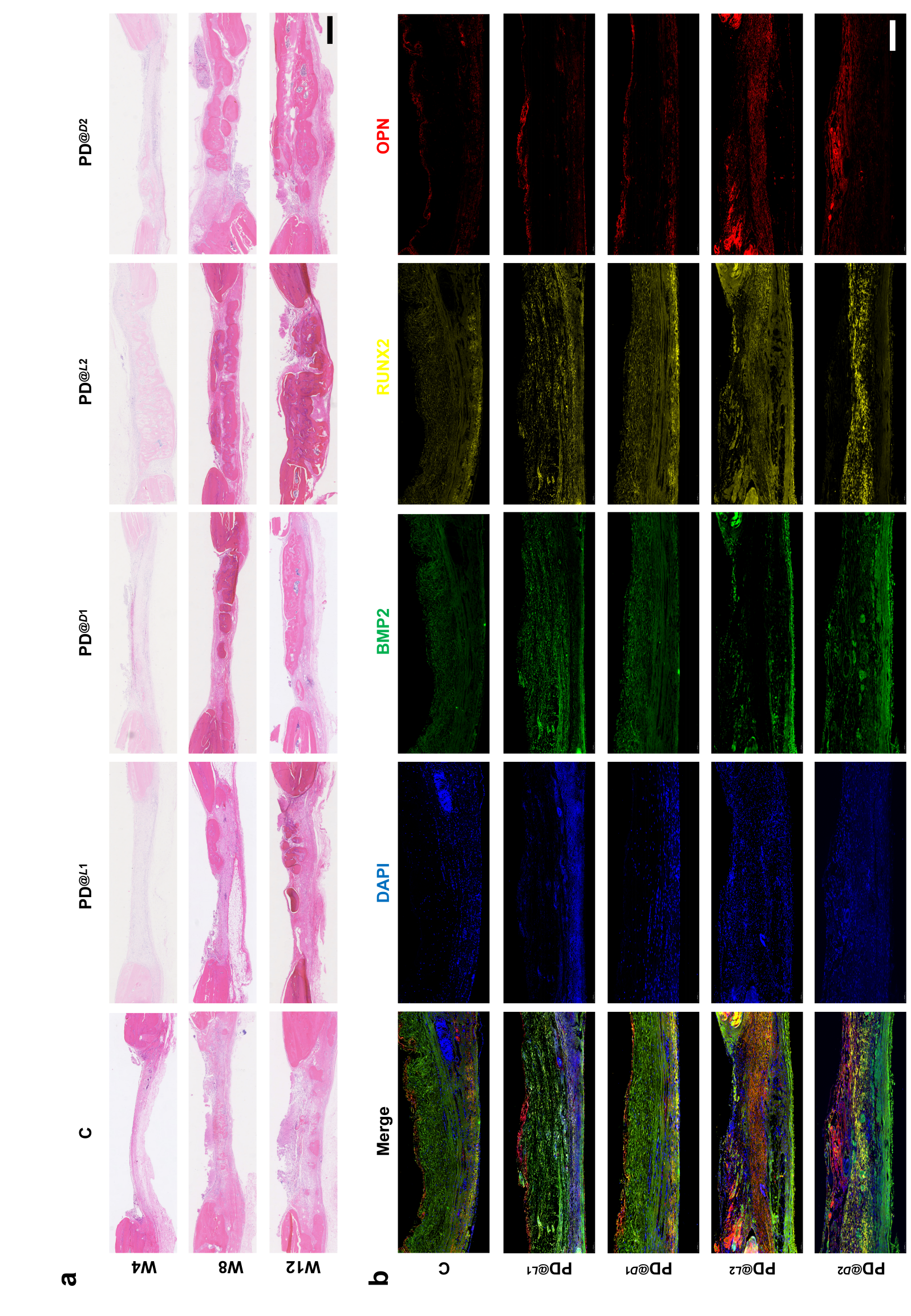


**Supplementary Figure 5.** Osteogenic marker expression in rat calvarial defect regions.
a) Representative histological sections of the defect site stained with H&E (scale bar: 500 µm). b) Immunofluorescence localization of BMP2/RUNX2/OPN in regenerated bone at 4 weeks post-implantation (scale bar: 500 µm).


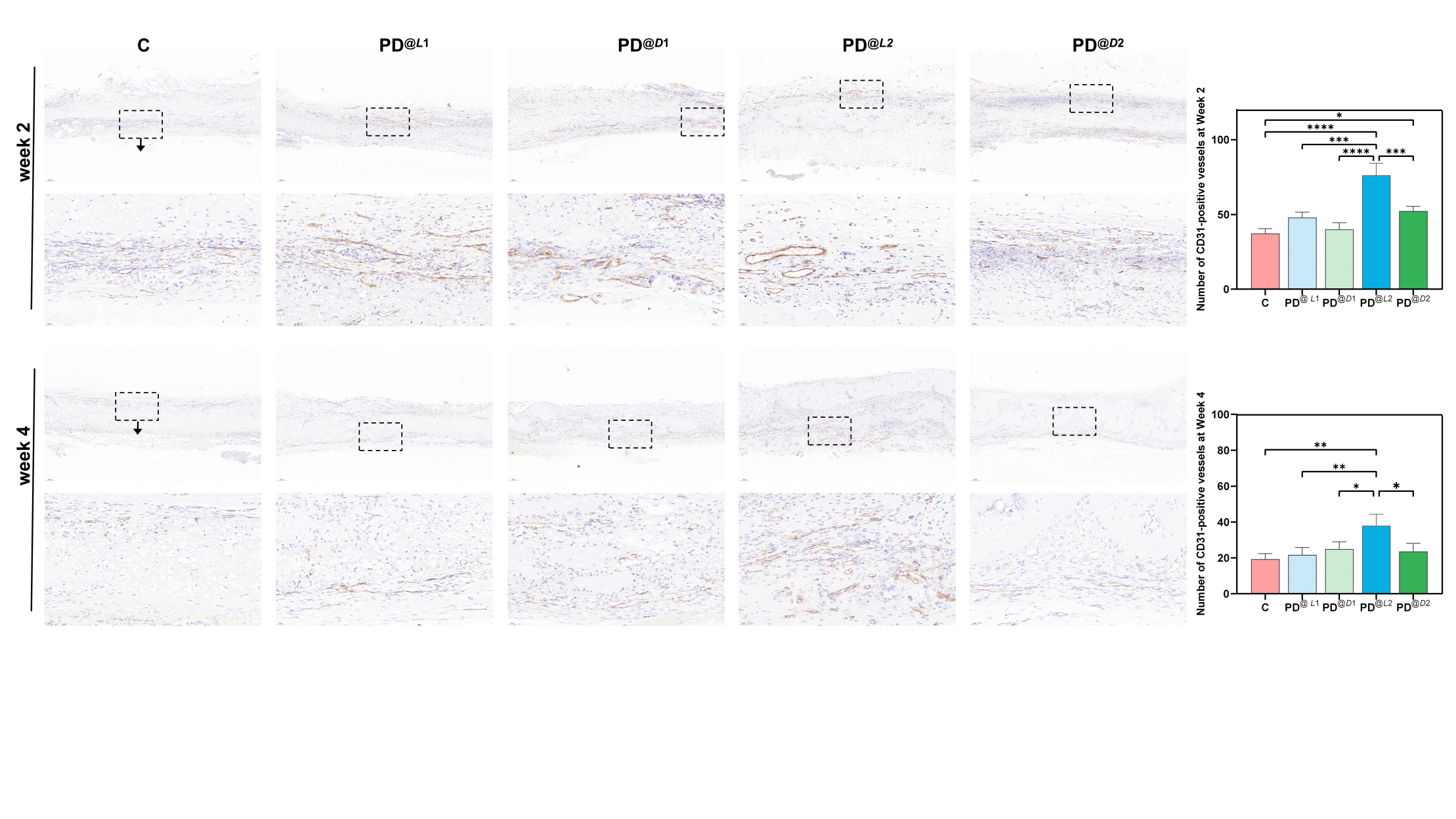


**Supplementary Figure 6.** Angiogenic marker expression in rat calvarial defect regions.

Representative photomicrographs and quantitative analysis of CD31 immunohistochemistry in the regenerated bone at 2 and 4 weeks post-implantation.

# *In vivo* degradation and cell retention

#
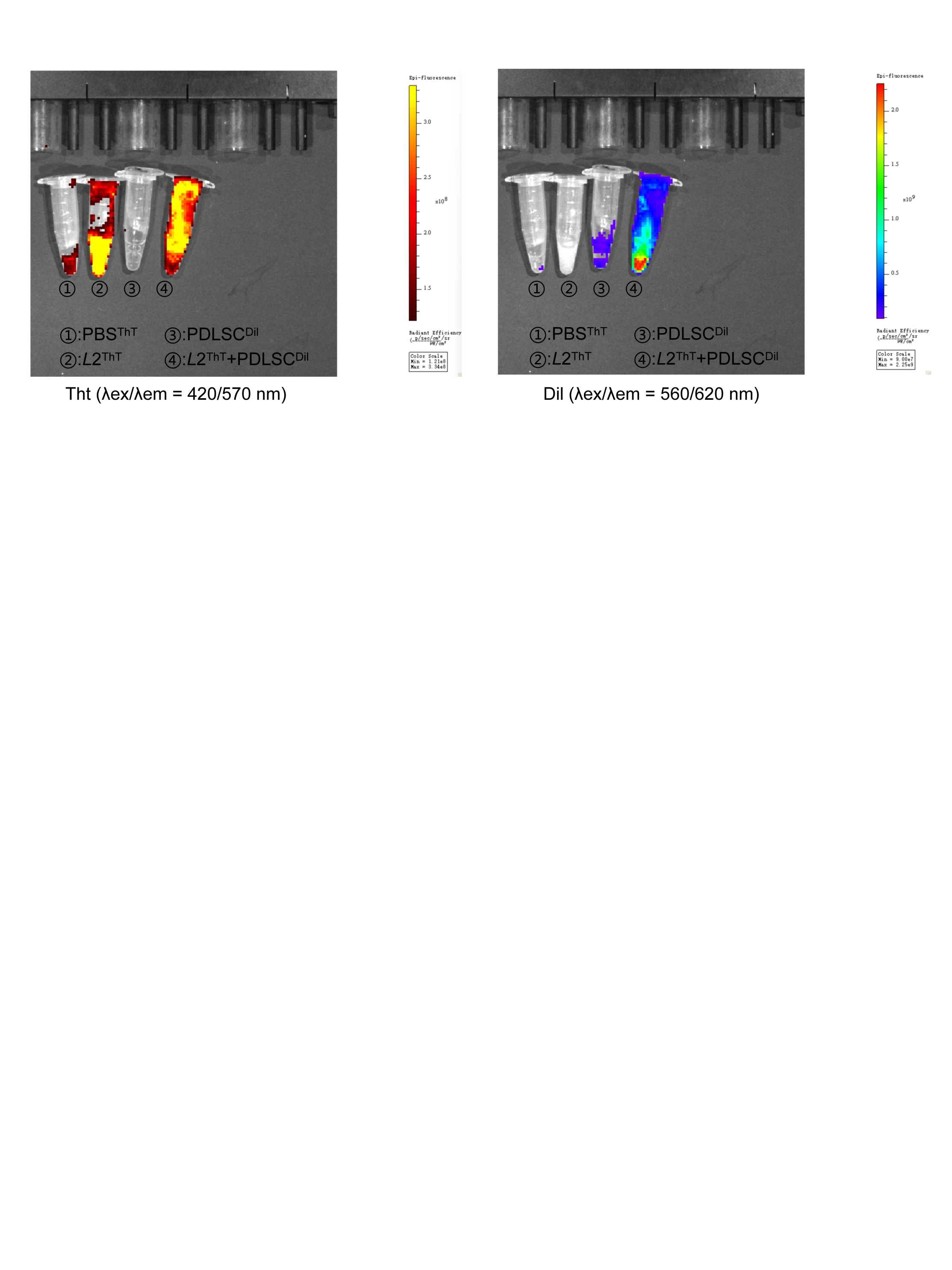


**Supplementary Figure 7.** Distinct Fluorescence Signals Enable Simultaneous Monitoring of Biomaterials and Stem Cells.

*In vitro* fluorescence imaging of implants from the four indicated groups: PBS^ThT^, *L*2^ThT^, PDLSC^Dil^, and *L*2^ThT^+PDLSC^Dil^. The *L2* fibers and PDLSCs were pre-labeled with Thioflavin T (ThT, λex/λem = 420/570 nm) and Dil (λex/λem = 560/620 nm), respectively. Fluorescence quantification verified ThT signal enhancement upon binding to the *L2* fibers. The minimal spectral overlap between ThT and Dil channels enabled clear signal discrimination and co-localization analysis *in vivo*.

#
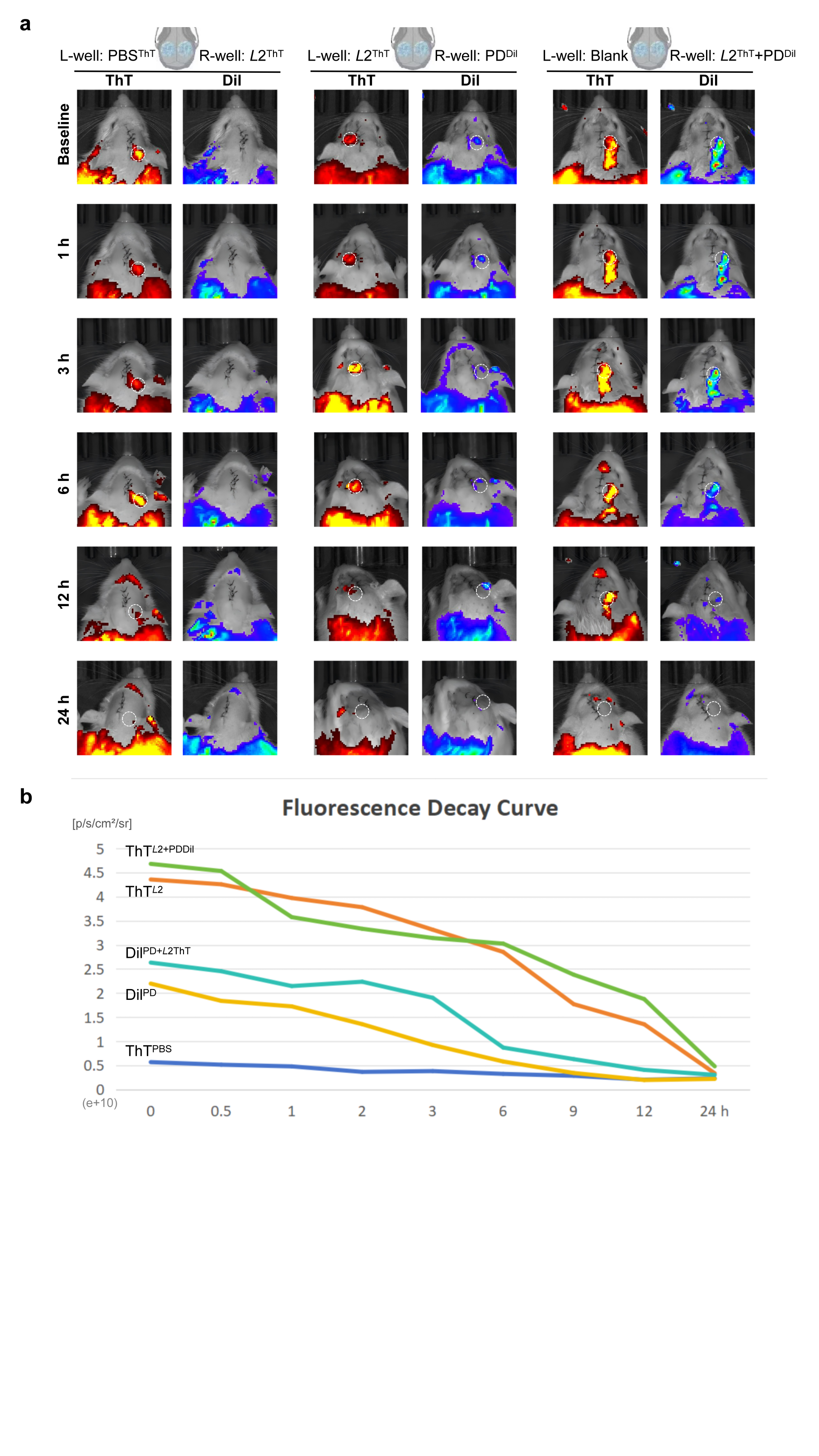


**Supplementary Figure 8.** *In vivo* tracking of chiral fibril degradation and PDLSC retention.

Fluorescence signals from ThT-labeled chiral fibrils and DiI-labeled PDLSCs were monitored over 24 hours post-implantation. The ThT signal associated with *L*2 fibrils was significantly more intense than the PBS control and exhibited a steady decline. The DiI signal from PDLSCs became markedly weak by 9 hours, with both signals largely diminished by 24 hours, indicating near-complete degradation and clearance.

# Biosafety validation in vivo

**
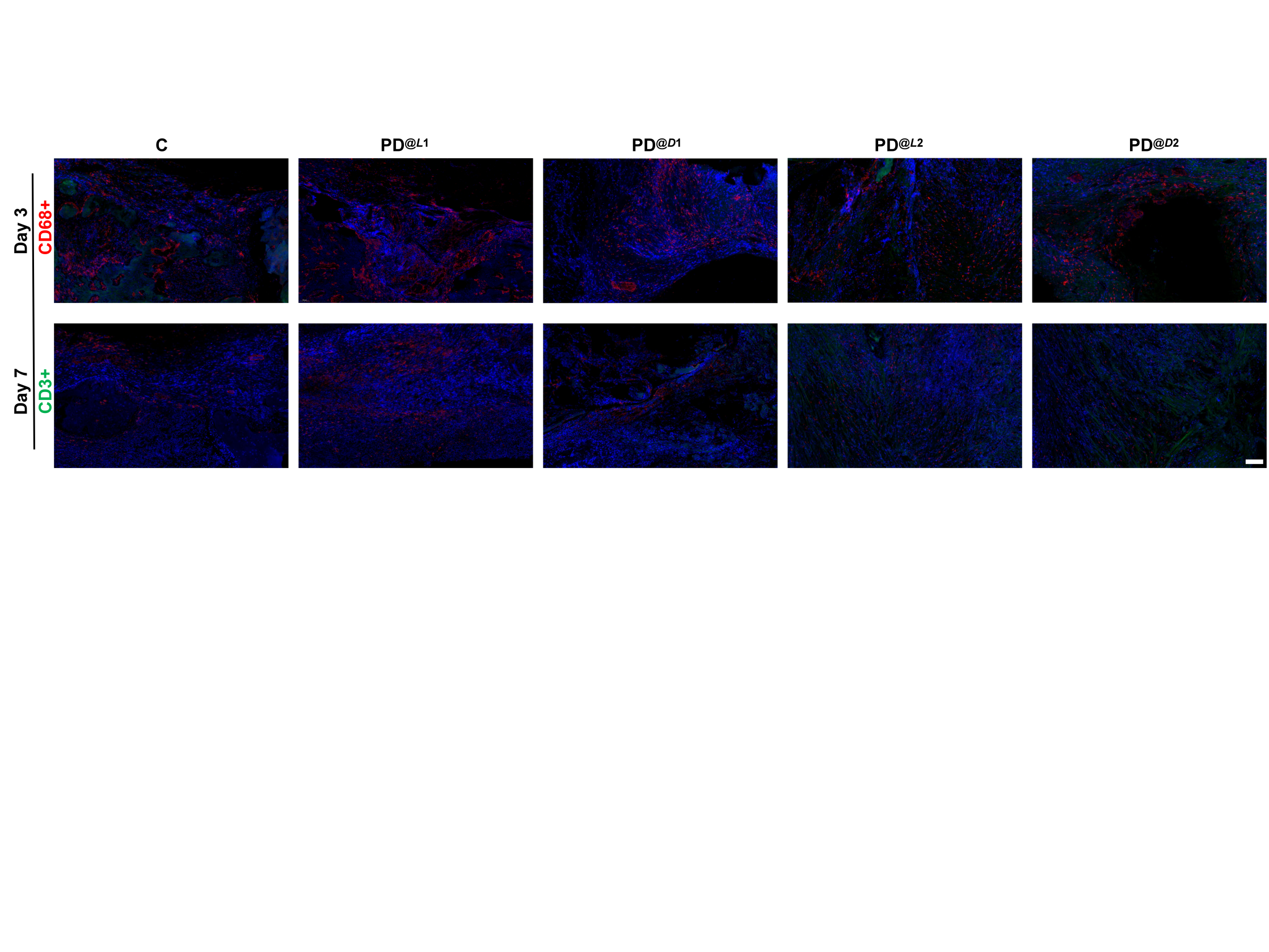
Supplementary Figure 9.** Representative immunofluorescence images showing the host immune response to the implant at the skull defect site at 3 and 7 days post-operation.

T lymphocytes and macrophages were stained for CD3 (green) and CD68 (red), respectively. Cell nuclei were counterstained with DAPI (blue). Scale bar: 100 µm.


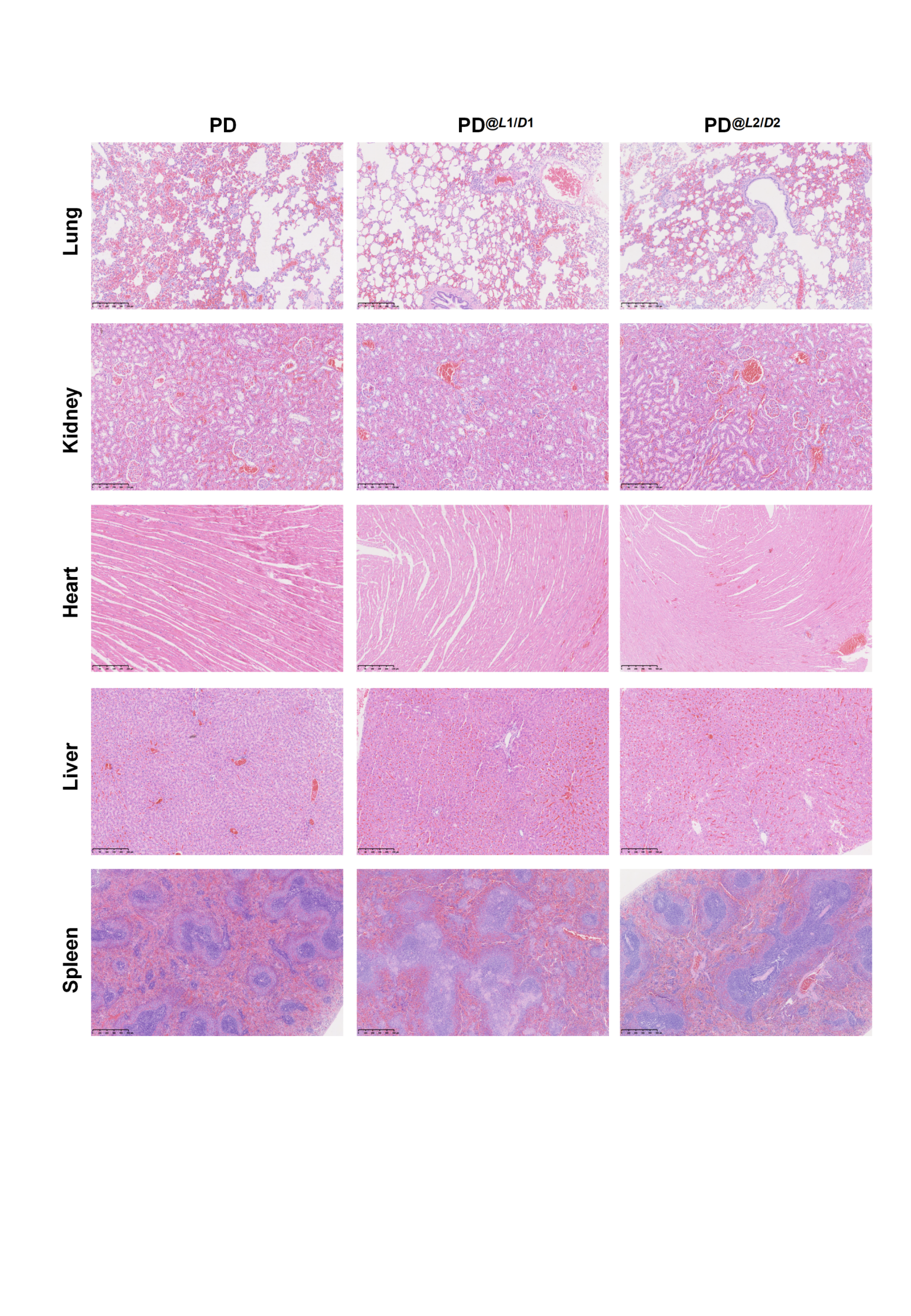


**Supplementary Figure 10.** Biosafety evaluation of chiral fibrils in rats.

The chiral material-PDLSCs mixture was implanted into rats, and major organs (heart, liver, spleen, lungs, and kidneys) were harvested at 4 weeks post-implantation for histological analysis. Hematoxylin and eosin (H&E) staining revealed no significant inflammatory infiltration or necrotic lesions, indicating excellent biocompatibility of the chiral material system.

# Materials and methods *in vitro*

**Cell Culture**

The human primary cells utilized in this study included human periodontal ligament stem cells (PDLSCs; Pricella, CP-H234) and human umbilical vein endothelial cells (HUVECs; Pricella, CP-H082), which were obtained from Procell Life Science & Technology Co., Ltd. (Wuhan, China). hPDLSCs were maintained in α-MEM medium (Hyclone, SH30265.01) supplemented with 10% (v/v) fetal bovine serum (FBS; Procell, 164210-50) and 1% (v/v) penicillin-streptomycin (Procell, PB180120), while HUVECs were cultured in endothelial cell medium (ECM; ScienCell, 1001) with identical supplements. All cells were incubated at 37°C within a humidified atmosphere containing 5% CO₂.

For optimal stimulation concentration determination, cells were exposed to chiral nanofibers at graded concentrations (10-50 μg/mL). Cell viability was assessed using CCK-8 assays (Dojindo, CK04), with live/dead cell discrimination further confirmed by Calcein-AM/PI double staining (Thermo Fisher, L3224/P3566). Pathway validation was performed using key protein inhibitors co-cultured with cells, including the Itgα5β1 antagonist Cilengitide (MCE, HY-16141), the cytoskeletal inhibitor Latrunculin A (Solarbio, IL1980), and the Piezo1 inhibitor GsMTx4 (MCE, HY-P1410).

Conditioned media (CM) were prepared by culturing hPDLSCs with chiral nanofibers for 72 hours, after which the supernatant was collected and centrifuged at 300 × g for 5 minutes to remove cellular debris followed by sterile filtration through a 0.22 μm membrane, and the resulting CM was then used to culture HUVECs for 24 hours before performing angiogenic assays ^4^.

***In Vitro* Assays**

CCK-8 Cell Viability Assay: Cell viability was assessed using the CCK-8 assay following co-culture of cells with chiral materials at various concentrations. At predetermined time points, the culture medium was aspirated, and 10% (v/v) CCK-8 enhancement solution (Tongren) was added to the wells. The cells were then incubated in a cell culture incubator. After a reaction time of 1–4 hours, the absorbance values at a wavelength of 450 nm were measured using a microplate reader, and the cell survival rate was calculated.

Live/Dead Cell Staining: Live/dead cell staining was performed after co-culture of cells with chiral materials for a specified duration. The Calcein-AM/PI dual-staining kit (Beyotime) was used to prepare the working solution, which was added to the cell culture wells and incubated at 37°C in the dark for 15 minutes. Following incubation, the cells were washed with PBS (Beyotime) and observed under a fluorescence microscope to distinguish live cells (green) from dead cells (red).

Transcriptomics and Pathway Analysis: Transcriptome sequencing analysis was conducted on periodontal ligament stem cells (PDLSCs) treated with *L*2 for 2 hours and an untreated control group. Total RNA was extracted using the Trizol method and assessed for integrity using an Agilent 2100 Bioanalyzer (RIN ≥ 7). RNA sequencing libraries were constructed using the NEBNext Ultra II RNA Library Prep Kit and sequenced on the Illumina NovaSeq 6000 platform with 150 bp paired-end sequencing. The raw sequencing data were subjected to quality control using Trimmomatic and aligned to the human reference genome (GRCh38) using HISAT2. Differential expression analysis was performed using DESeq2 software with a screening criterion of |log2FC| ≥ 1 and FDR < 0.05. Differentially expressed genes were annotated using DAVID and subjected to GO pathway enrichment analysis. To validate the sequencing results, selected differentially expressed genes were verified by RT-qPCR, and the expression levels of key pathway-related proteins were assessed via Western blot analysis.

Real-Time Quantitative Reverse Transcription Polymerase Chain Reaction (RT-qPCR): (i) RNA Extraction: Cells were lysed using Trizol reagent (Invitrogen, USA), and the aqueous phase was collected following phase separation with chloroform. RNA was precipitated with isopropanol, washed with 75% (v/v) ethanol, and dissolved to determine its concentration. (ii) cDNA Synthesis: One microgram of RNA was reverse-transcribed using a reverse transcription kit (Takara Bio Inc., Japan) with the following protocol: 25°C for 10 minutes, 45°C for 15 minutes, and 85°C for 5 seconds. (iii) qPCR Detection: A 20-μL reaction mixture containing SYBR Green Mix (Roche, Germany), primers (0.5 μM each), and cDNA template (10 ng) was prepared (the primer sequences are listed in Supplementary Table 1). The thermal cycling conditions were as follows: initial denaturation at 95°C for 30 seconds, followed by 40 cycles of 95°C for 5 seconds and 60°C for 30 seconds (Applied Biosystems, Foster City, CA, USA). Melting curve analysis was performed to confirm the specificity of the amplification products. The relative gene expression levels were calculated using the 2^-ΔΔCt method with GAPDH as the internal housekeeping reference gene.

Western Blot Analysis: (i) Protein Extraction: Cells were lysed on ice with RIPA lysis buffer (Beyotime, Shanghai, China) with protease inhibitor cocktail (ThermoFisher, Rockford, Illinois, USA) for 30–60 minutes, followed by sonication. The lysates were centrifuged at 12,000–15,000×g for 15–20 minutes, and the supernatant was collected to determine protein concentration using a BCA kit (ThermoFisher, Rockford, Illinois, USA). (ii) Electrophoresis and Membrane Transfer: Equal amounts of protein were denatured and loaded onto an SDS-PAGE gel (160 V, 45 minutes) and transferred to a PVDF membrane at a constant current of 200 mA for 120 minutes. (iii) The membrane was blocked with 5% (w/v) skimmed milk for 1–2 hours at room temperature, followed by overnight incubation at 4°C with primary antibodies against FN (Abcam, ab268020), Itgα5 (Abcam, ab179483), Itgβ1 (HUABIO, ER31001), OPN (Abcam, ab307994), ALP (Abcam, ab307726), BMP2 (Abcam, ab284387), Vinculin (Abcam, ab129002), ERK1/2 (HUABIO, ET1601-29), p-Erk1/2 (HUABIO, ET1610-13), FAK(HUABIO, ET1602-25), p-FAK(HUABIO, ET1610-34) and GAPDH (Abcam, ab8245). After washing with TBST, the membrane was incubated with HRP-conjugated secondary antibodies (Goat Anti-Rabbit IgG, A0208; Goat Anti-Mouse IgG, A0216) at room temperature for 1 hour. (iv) Detection and Analysis: The proteins were visualized using chemiluminescent substrate, and images were captured using an imaging system. The expression levels of target proteins were quantified by densitometry analysis.

Alizarin Red S (ARS) and Alkaline Phosphatase (ALP) Staining Assays: PDLSCs were co-cultured with four groups of chiral HAPs materials in 24-well plates, with ALP staining performed on day 7 and ARS staining on day 21. Cells were gently washed with PBS to remove metabolic waste, fixed with 4% (w/v) paraformaldehyde at room temperature for 15 minutes, and then rinsed three times with PBS. Cells were stained with 0.2% (w/v) ARS solution (pH 8.3, Solarbio) for 5 minutes, and calcium nodules were observed as orange-red deposits under light microscopy. The BCIP/NBT working solution (Beyotime, prepared with 10 μL BCIP + 20 μL NBT + 3 mL ALP staining buffer) was applied to the fixed cells and incubated at 37°C in the dark for 30 minutes. The reaction was terminated with ddH₂O, and images were captured.

Spheroid Outgrowth Assay: (i) Agarose Mold Preparation: A 2% (w/v) agarose solution (1 g/50 mL ddH_2_O) was prepared, dissolved in a microwave, sterilized by autoclaving, and 700 μL was pipetted into molds (Microtissues®) while still warm. After solidification, the molds were transferred to 6-well plates. (ii) Spheroid Culture: Cells from T25 culture flasks were resuspended in 180 μL of culture medium, allowed to settle for 5–10 minutes, and then covered with 4 mL of culture medium over the molds. After 8–9 hours, the spheroids were collected. (iii) Collagen Gel Preparation: A collagen gel solution was prepared by mixing 670 μL of Type I collagen (Corning®, USA), 100 μL of 10 × PBS, 17 μL of 1 M NaOH, and 213 μL of H₂O. A final solution of 3 mL was obtained by combining 320 μL of FBS and 1,680 μL of ECM. Three hundred microliters of collagen solution was added to each well of a 24-well plate and allowed to solidify at 37°C for 10 minutes. Seven to eight spheroids per well were mixed with 300 μL of collagen solution, gently mixed, and distributed into three replicate wells. (iv) Result Observation: The number and length of outgrowths from 10 spheroids per group were quantified under a microscope after 12 and 24 hours of culture at 37°C.

Matrigel Tube Formation Assay: Corning Matrigel Basement Membrane Matrix (Becton-Dickinson and Company, Franklin Lakes, NJ, USA) was thawed and added to pre-chilled 48-well plates at a volume of 100 μL per well. The plates were incubated at 37°C for 30 minutes to allow the Matrigel to solidify. Single-cell suspensions (5 × 10⁵ cells/mL) from each group were prepared and seeded onto the Matrigel-coated wells. Images were captured under an inverted microscope at 4 and 8 hours of culture, and vascular formation parameters, including total length, segment number, node number, and mesh number, were analyzed using Image-pro plus 6.0 software.

Immunofluorescence Staining: PDLSCs were seeded in 24-well confocal culture dishes at a density of 5 × 10⁴ cells per well. Following fixation with 4% (w/v) paraformaldehyde and permeabilization with 0.1% (v/v) Triton X-100, the cells were blocked with 3% (w/v) bovine serum albumin (BSA) for 1 h at room temperature. Subsequently, the samples were incubated overnight at 4°C with the following primary antibodies: Itgα5 (Abcam, ab150361), Itgβ1 (Abcam, ab30394), HIF-1α(HUABIO, HA721997), BMP2 (Abcam, ab284387) and RUNX2 (Abcam, ab192256). After thorough washing, the cells were probed with appropriate fluorescently-labeled secondary antibodies for 1 h at room temperature in the dark. Cell nuclei were stained with DAPI, and the slides were mounted. Images were acquired using a laser confocal microscope (Olympus inverted confocal FV3000), and fluorescence intensity was quantified using ZEN software. Negative controls and at least three replicates were included to ensure the reliability of the results.

Calcium Influx Detection (Fluo-4 AM Method): Intracellular Ca² concentration changes were monitored using the Fluo-4 AM fluorescent probe (Beyotime, S1060). Cells were incubated with 2 μM probe in the dark for 30 minutes, washed with HBSS buffer, and then incubated at 37°C for 20 minutes to facilitate esterase hydrolysis. The cell nuclei were stained with Hoechst 33342 staining solution for live cells (Beyotime, C1028) for 10 minutes at room temperature, followed by three washes with phosphate-buffered saline (PBS). Fluorescence intensity was assessed using a laser confocal microscope with appropriate excitation wavelength.

**Statistical Analysis**

Data are presented as mean ± SEM. Differences among more than two groups were analyzed by one-way ANOVA, followed by the Holm-Šidák post hoc test for multiple comparisons. Comparisons between two unpaired groups were performed using an unpaired two-tailed Student's t-test. A *P* value of less than 0.05 was considered statistically significant.

# Biocompatibility Assessment of PDLSCs and HUVECs


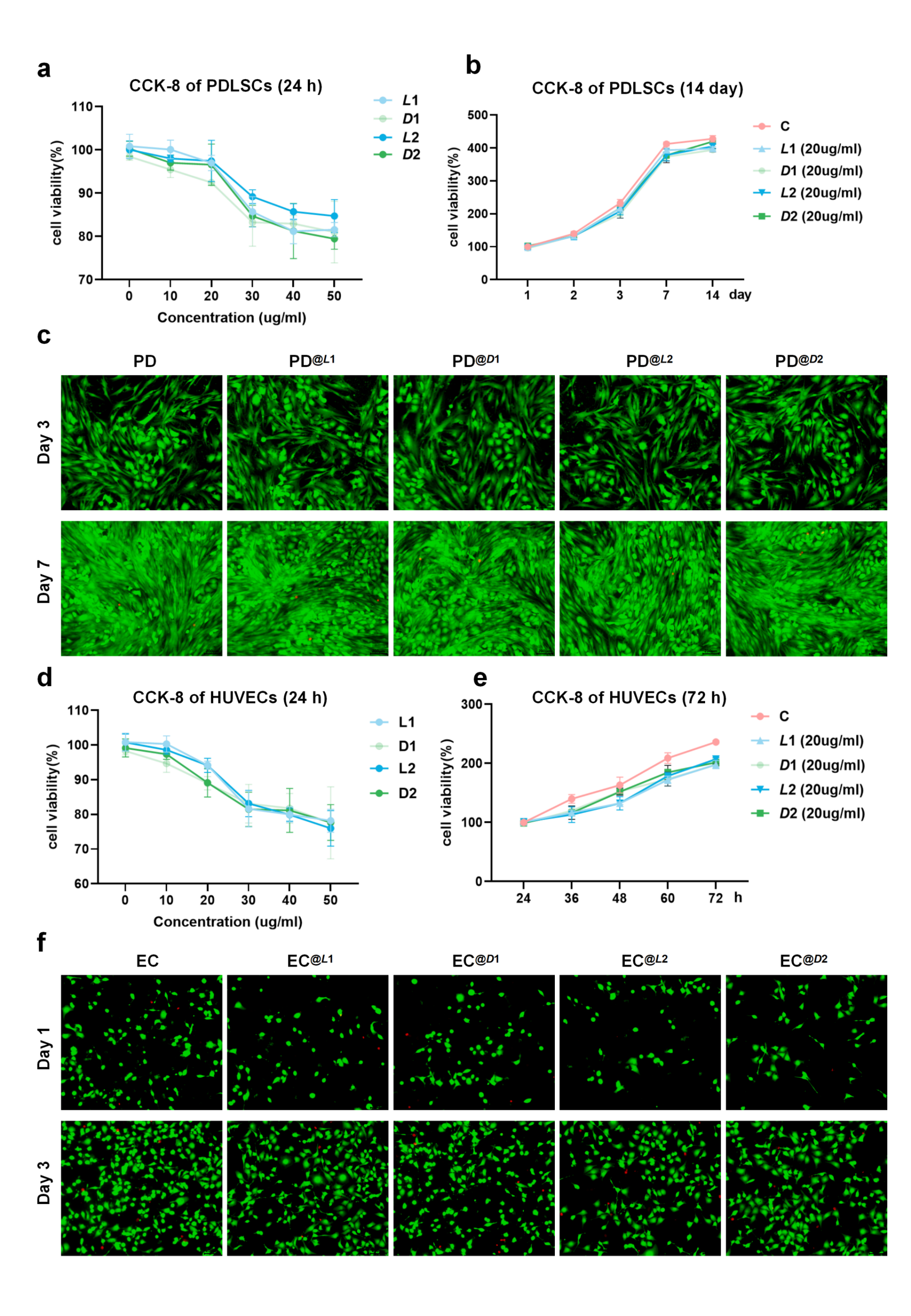


**Supplementary Figure 11.** Cytocompatibility profiling and dosage optimization of chiral nanomaterials.
a) CCK-8 viability screening of PDLSCs across concentration gradients (0-50 µg/mL, 24h). b) Longitudinal viability monitoring at 20 µg/mL over 14 days. c) Calcein-AM/PI viability staining (green/red fluorescence) at 20 µg/mL after 3/7-days of culture (scale bars: 200 µm). d) CCK-8 viability screening of HUVECs across concentration gradients (0-50 µg/mL, 24 h). e) Longitudinal viability monitoring of HUVECs at 20 µg/mL for 72 h. f) Live/Dead staining (Calcein-AM/PI) of HUVECs cultured with 20 µg/mL for 1/3 days (green: viable cells; red: non-viable cells; scale bars: 200 µm). Chiral materials exhibit excellent biocompatibility within therapeutic concentration ranges.

# Quantitative Analysis of Osteogenesis and Angiogenesis


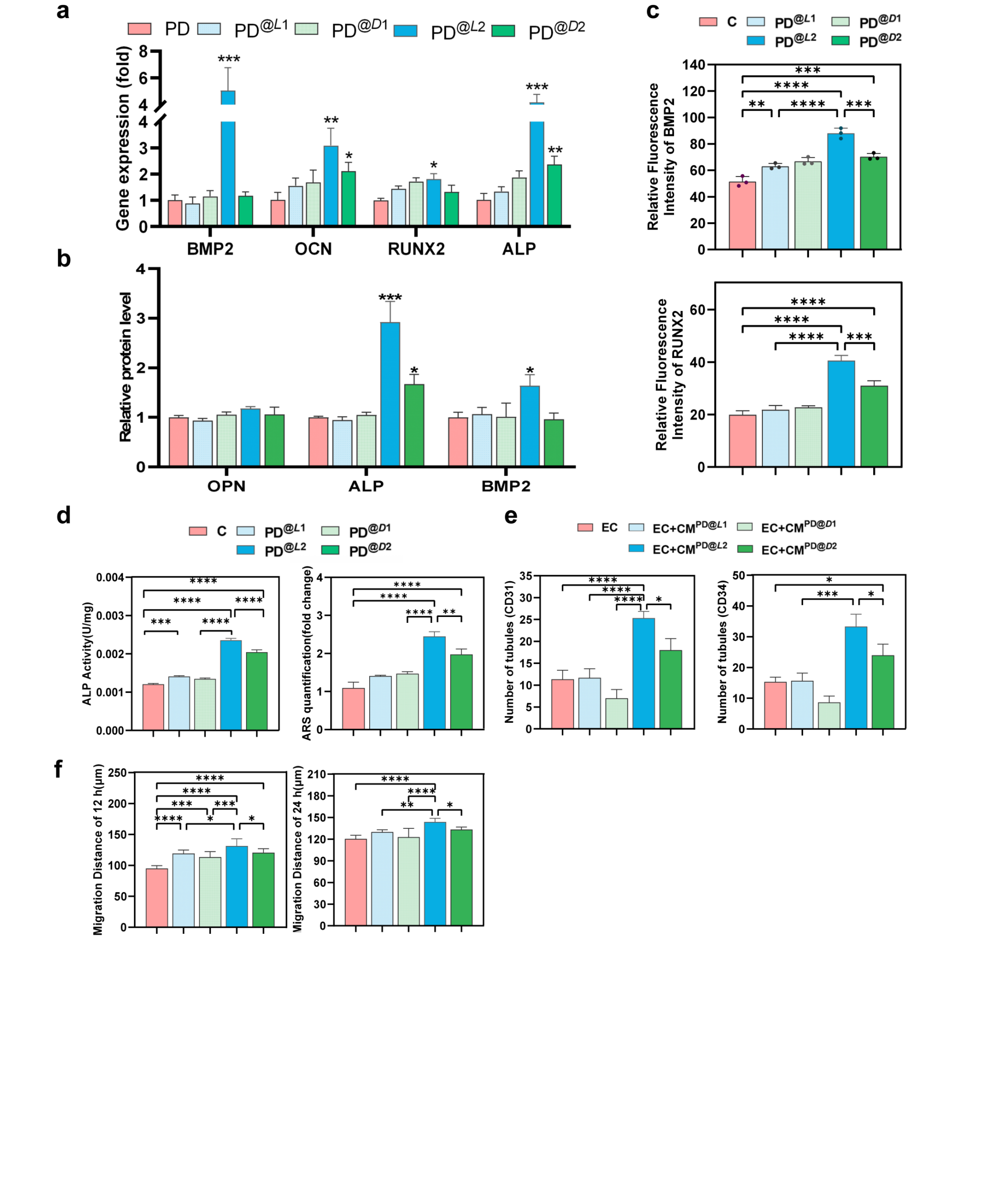


**Supplementary Figure 12.** Quantitative analysis of *L*2-enhanced osteogenic and angiogenic responses.
a) RT-qPCR quantification of osteogenic markers after 7-day stimulation (Data are presented as mean ± s.d.; **P* < 0.05, ** *P* <0.01, *** *P* <0.001 versus control; one-way ANOVA with Holm-Šidák post hoc test). b) Quantitative analysis of OPN, ALP, and BMP2 protein expression levels by Western blot. c) Statistical analysis of BMP2/RUNX2 immunofluorescence intensity. d) ALP/ARS staining quantification. e) Morphometric analysis of *in vivo* vascular structures, including endothelial marker (CD31/CD34) expression in newly formed tubules. f) Quantitative analysis of migration distance in spheroid sprouting assays at 12 and 24 hours (**P* < 0.05, ***P* < 0.01, ****P* < 0.001). *L*2 stimulation induces significant enhancements of both osteogenic and angiogenic parameters compared to controls.

# ALP Staining

#
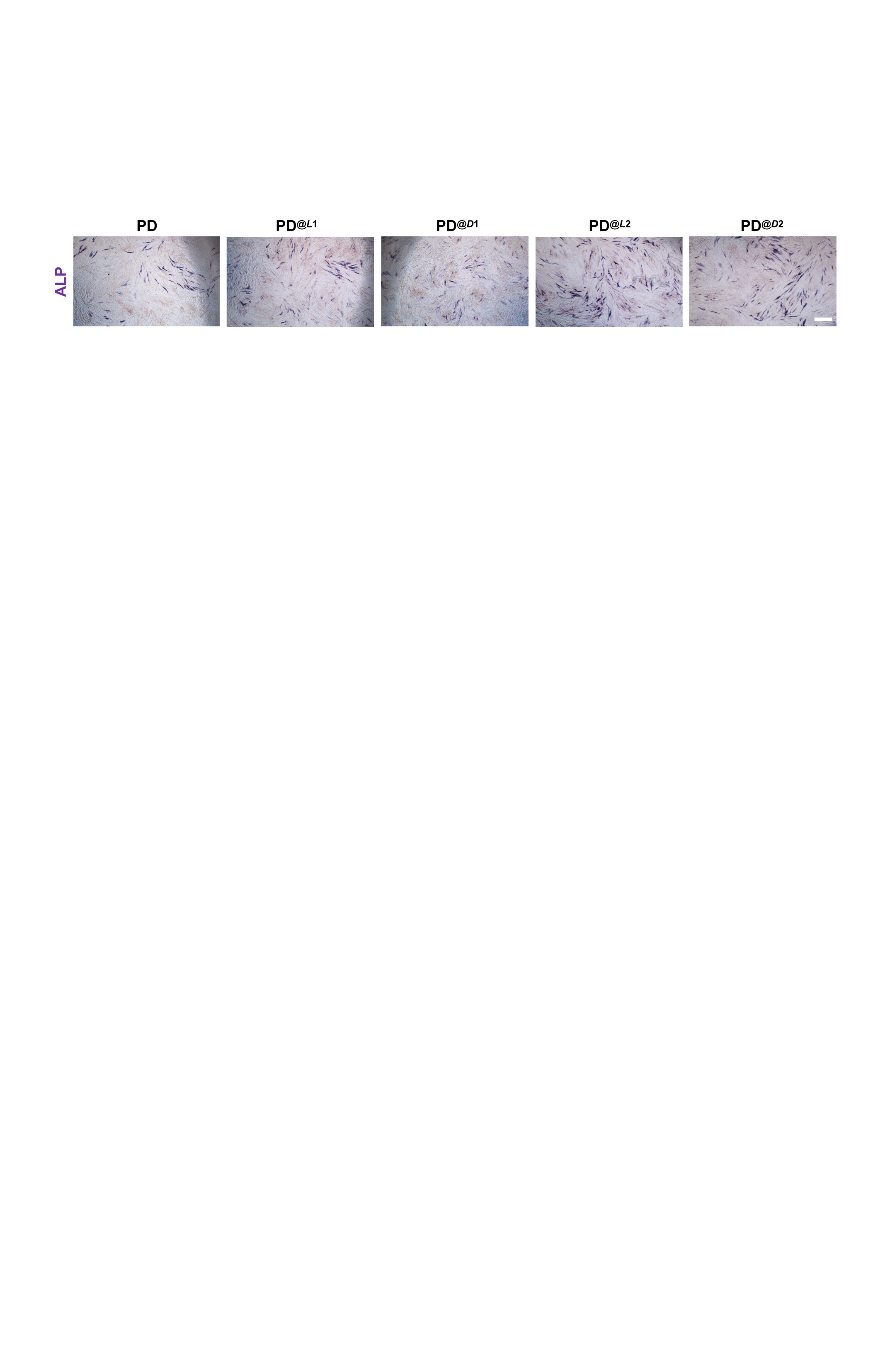


# **Supplementary Figure 13.** ALP activity assessment of PDLSCs at day 7 (scale bars: 500 µm).

# Chiral material-mediated angiogenesis in HUVECs


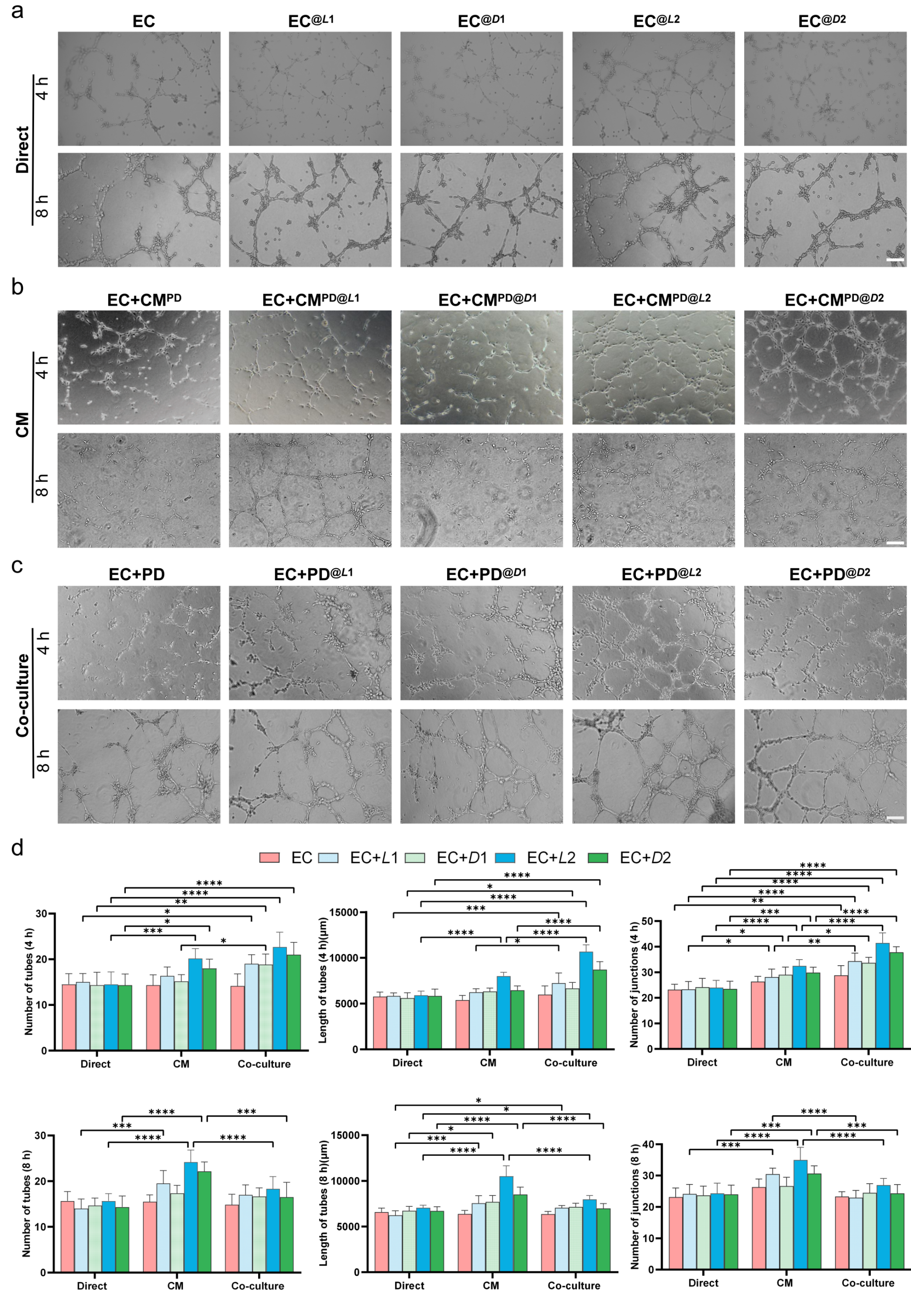


**Supplementary Figure 14.** Matrigel tube formation assay of HUVECs under different culture conditions.

1. Direct stimulation of HUVECs with the chiral fibrils. b) Indirect stimulation via conditioned medium from PDLSCs pre-treated with chiral fibrils. c) direct co-culture of HUVECs with PDLSCs at a ratio of 4:1 (Scale bar: 200 µm). d) Angiogenic parameters, including network complexity metrics (number of junctions, number of tubes, and length of tubes), were systematically quantified in the Matrigel tube formation assay at 4 and 8 hours. Data were analyzed by two-way ANOVA (**P* < 0.05, ***P* < 0.01, ****P* < 0.001).

# Chiral matrix-selective integrin activation

**Supplementary Figure 15.** Quantitative analysis of Western blot results for integrin-related proteins.


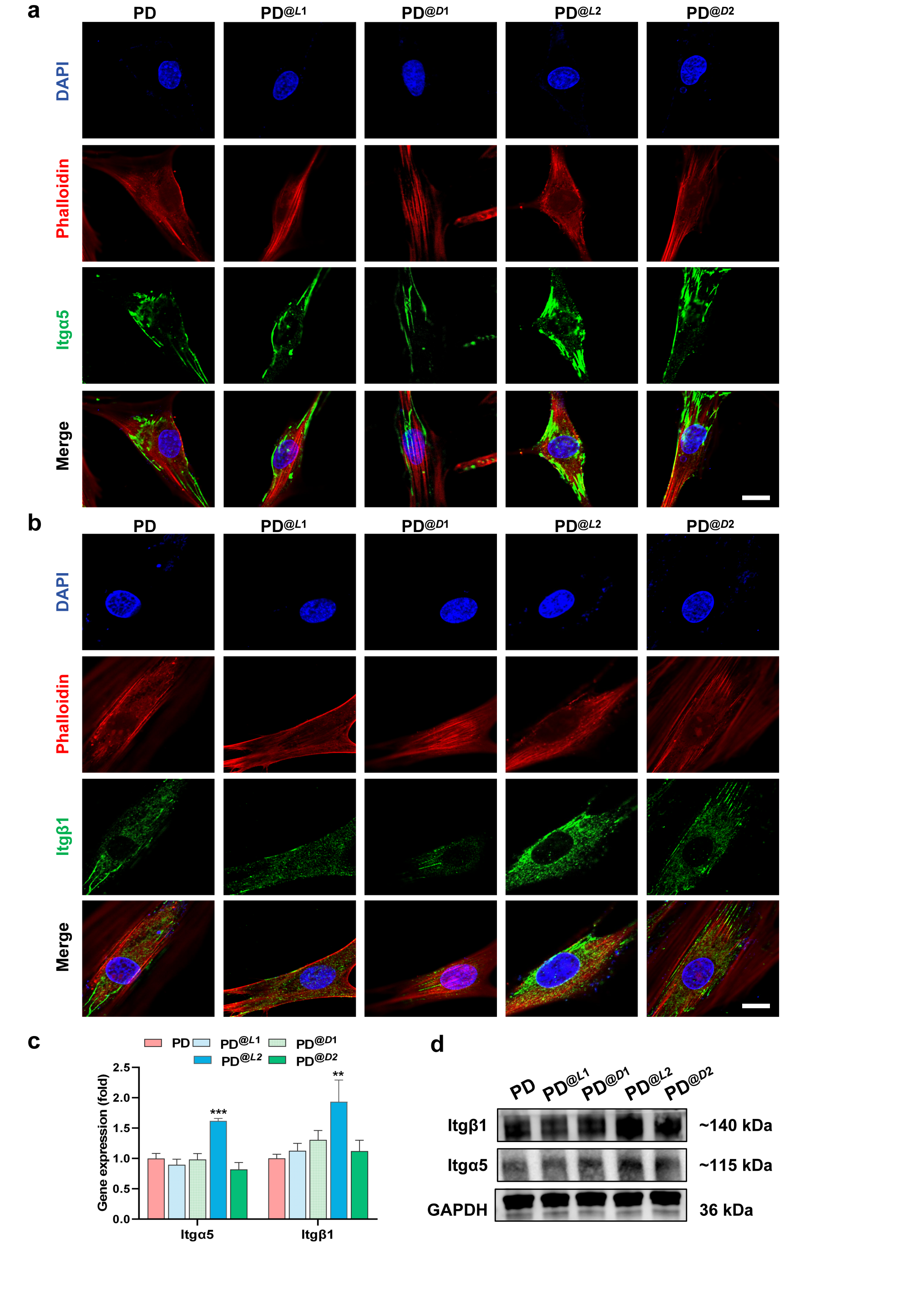


**Supplementary Figure 16.** Chiral matrix-selective activation of integrin heterodimers.

a-b) Immunofluorescence analysis demonstrating enhanced clustering of Itgα5β1 heterodimers at focal adhesion sites in the *L*2-treated groups versus control and other matrix conditions (scale bars: 10 µm). c) Quantitative qPCR profiling of integrin subtypes revealed significant upregulation of Itgα5 and Itgβ1 mRNA expression levels in *L*2-treated groups, while no statistically significant alterations were observed in other experimental groups (error bars indicate s.d., ***P* < 0.01, ****P* < 0.001 versus control, one-way AVONA analysis). d) A representative Western blot confirming the elevated expression of Itgα5β1 heterodimers in *L*2-treated samples.

# Inhibition of the Itg**α5β1**-cytoskeleton axis reverses *L*2-driven osteogenesis


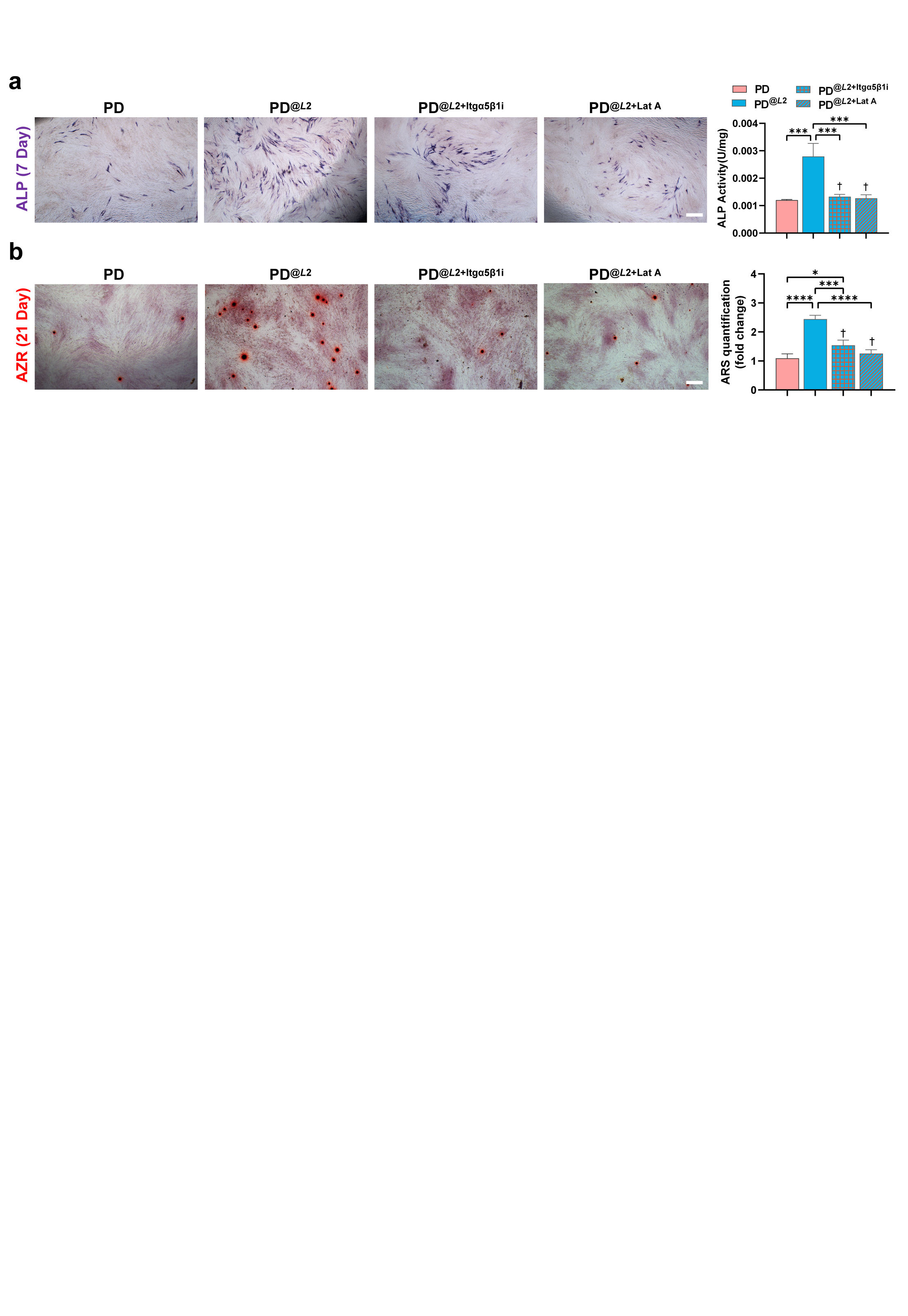


**Supplementary Figure 17.** Effects of *L*2 topography and signaling inhibition on the osteogenic differentiation of PDLSCs.

1. Alkaline phosphatase (ALP) staining and quantitative analysis of PDLSCs stimulated by *L*2 in the presence or absence of cilengitide (integrin α5β1 inhibitor) or Latrunculin A (Lat A, actin polymerization inhibitor); scale bar: 500 µm. **b)** Alizarin Red S (ARS) staining and quantitative analysis of mineralized nodule formation; scale bar: 500 µm. Data are presented as mean ± SD; **P* < 0.05, ****P* < 0.001, †*P* < 0.05 versus the PD^@^*^L^*^2^ group; one-way ANOVA.

# Reversal of *L*2-driven angiogenesis by inhibiting the Itg**α5β1**-cytoskeleton-Piezo1 signaling axis


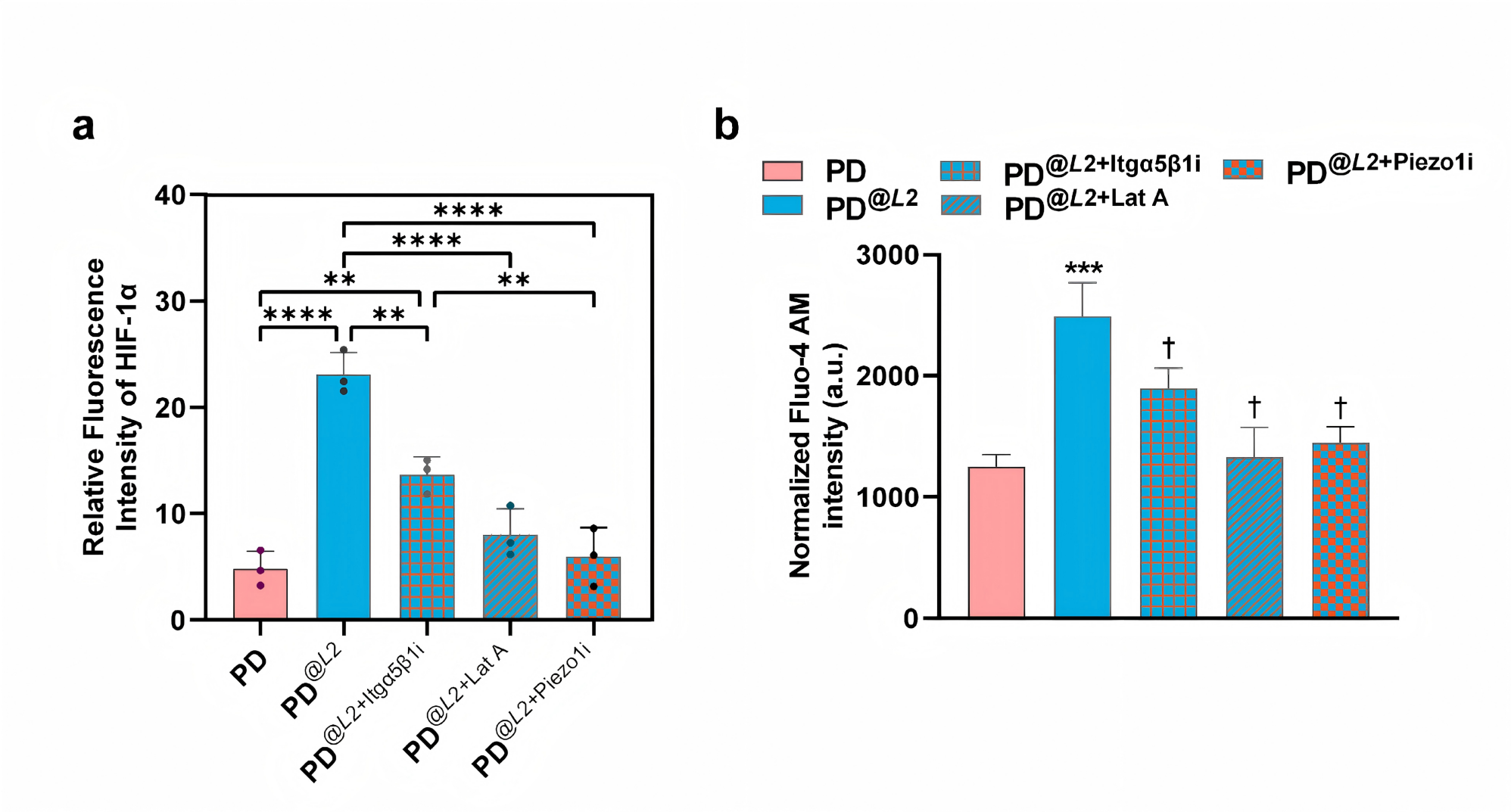


**Supplementary Figure 18.** a) Quantification of HIF-1α immunofluorescence intensity. b) Quantification of Fluo-4 AM fluorescence intensity reflecting intracellular Ca²⁺levels.

Differences among groups were analyzed by one-way ANOVA followed by the Holm-Šidák post hoc test. ***P* < 0.01, ****P* < 0.001 versus the PD group; †*P* < 0.05 versus the PD^@^*^L^*^2^ group.


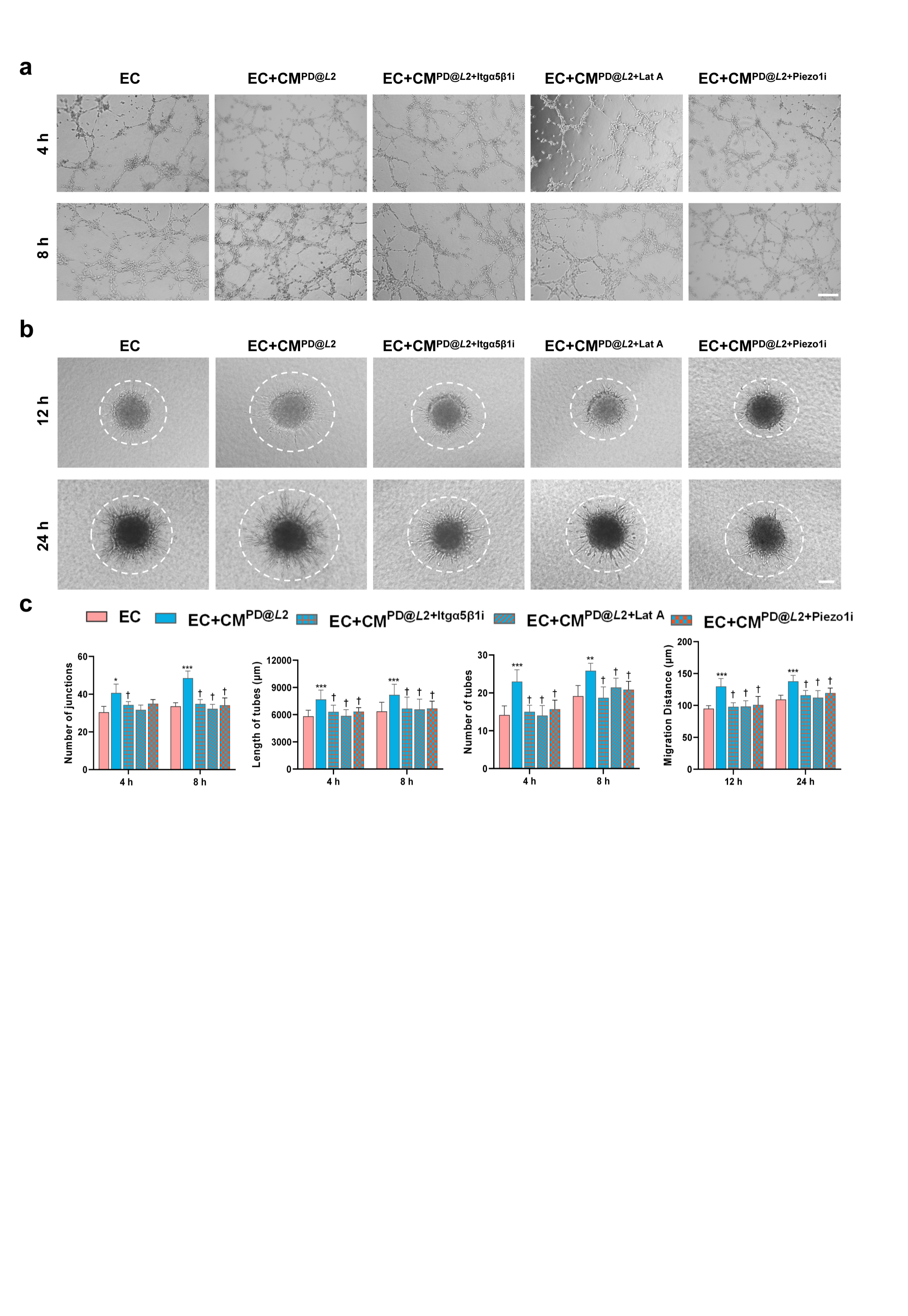


**Supplementary Figure 19. Angiogenic effects of PD^@^*^L^*^2^ conditioned medium under mechanosensitive receptor inhibition.**

a) Representative images of tube formation in endothelial cells cultured with conditioned medium from PD^@^*^L^*^2^‑treated cells, in the presence or absence of cilengitide (integrin α5β1 inhibitor), Latrunculin A (actin polymerization inhibitor), or GsMTx4 (Piezo1 inhibitor); Scale bar: 200 µm. b) Representative images of spheroid sprouting assay and quantified migration distance of endothelial sprouts; scale bar: 100 μm. c) Quantitative analysis of tube formation and spheroid sprouting assays. Error bars represent standard deviation (s.d.); **P* < 0.05, ***P* < 0.01,****P* < 0.001 versus the EC+CM^PD^ control group; †*P*<0.05 versus the EC+CM^PD@^*^L^*^2^ group; one-way ANOVA with Holm-Šidák post hoc test.

# Micro-CT Quantification of Defect Bone Height


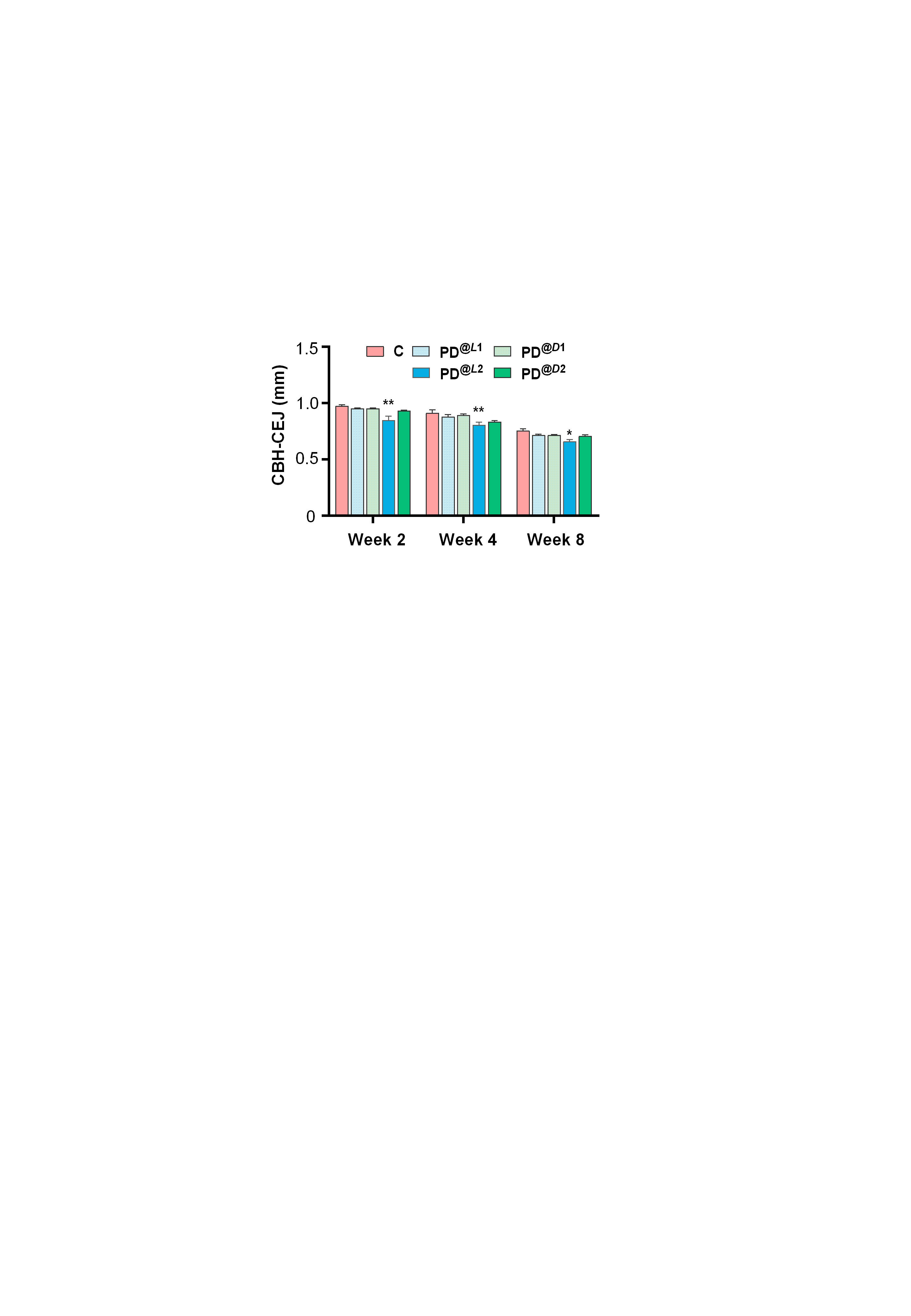


**Supplementary Figure 20.** Quantitative assessment of osseous regeneration based on Micro-CT results, showing dynamic changes in bone height within the defect region (**P* < 0.05, ***P* < 0.01 vs. control; one-way ANOVA with the Holm-Šidák post hoc test).

# Primer sequences of the gene markers analyzed by RT-qPCR

**Supplementary Table 1. Primer sequences of the gene markers analyzed by RT-qPCR experiments.**

| Gene Symbol | 5’ - 3’ |
| --- | --- |
| FN1 (human) F | GGCCACACCTACAACCAGTA |
| FN1 (human) R | TCGTCTCTGTCAGCTTGCAC |
| ITGA5 (human) F | GCCTGTGGAGTACAAGTCCTT |
| ITGA5 (human) R | AATTCGGGTGAAGTTATCTGTGG |
| ITGB1 (human) F | CTACTTCTGCACGATGTGATGAT |
| ITGB1 (human) R | TTGGCTGGCAACCCTTCTTT |
| GAPDH (human) F | ACCCAGAAGACTGTGGATGG |
| GAPDH (human) R | CACATTGGGGGTAGGAACAC |
| VEGFA (human) F | GTGAGGTTTGATCCGCATGAT |
| VEGFA (human) R | GACCCTGGCTTTACTGCTGTA |
| GLUT1(human)F | CTTTTCTGTTGGGGGCATGAT |
| GLUT1(human)R | CCGCAGTACACACCGATGAT |
| PDK1(human)F | GGAGGTCTCAACACGAGGTC |
| PDK1(human)R | GTTCATGTCACGCTGGGTAA |
| BMP2 (human) F | ACCCGCTGTCTTCTAGCGT |
| BMP2 (human) R | TTTCAGGCCGAACATGCTGAG |
| RUNX2 (human) F | TGGTTACTGTCATGGCGGGTA |
| RUNX2 (human) R | TCTCAGATCGTTGAACCTTGCTA |
| OCN (human) F | GGCAGCGAGGTAGTGAAGAG |
| OCN (human) R | GATGTGGTCAGCCAACTCGT |
| ALP (human) F | AAGGCTTCTTCTTGCTGGTG |
| ALP (human) R | GCCTTACCCTCATGATGTCC |

# References:

1 Subbiah, R. *et al.* Prevascularized hydrogels with mature vascular networks promote the regeneration of critical-size calvarial bone defects in vivo. *J Tissue Eng Regen Med* **15**, 219-231, doi:10.1002/term.3166 (2021).

2 Wang, Y. *et al.* Mitochondria Transplantation to Bone Marrow Stromal Cells Promotes Angiogenesis During Bone Repair. *Adv Sci (Weinh)* **11**, e2403201, doi:10.1002/advs.202403201 (2024).

3 Babo, P. S. *et al.* Evaluation of a platelet lysate bilayered system for periodontal regeneration in a rat intrabony three-wall periodontal defect. *J Tissue Eng Regen Med* **12**, e1277-e1288, doi:10.1002/term.2535 (2018).

4 Iwasaki, K. *et al.* Angiogenic Effects of Secreted Factors from Periodontal Ligament Stem Cells. *Dent J (Basel)* **9**, doi:10.3390/dj9010009 (2021).
